# Welcome to paradise: Measuring vegetation responses to tourism and agricultural activity using Leaf Area Index in the Galapagos Islands

**DOI:** 10.1371/journal.pone.0344628

**Published:** 2026-06-10

**Authors:** Lauren A. Rhodes, Julio E. Tinoco, Gonzalo E. Sánchez, Bernard Moscoso

**Affiliations:** 1 Facultad de Ciencias Sociales y Humanísticas, Centro de Vinculación e Investigaciones Rurales, Escuela Superior Politécnica del Litoral, ESPOL, Guayaquil, Ecuador; 2 Facultad de Ciencias Sociales y Humanísticas, Centro de Investigaciones Económicas, Escuela Superior Politécnica del Litoral, ESPOL, Guayaquil, Ecuador; 3 Facultad de Estudios Internacionales, Universidad Espíritu Santo, Samborondón, Ecuador; City College of New York, UNITED STATES OF AMERICA

## Abstract

The Galapagos Islands are globally recognized for their biodiversity, yet the high volume of annual visitors and dependence on food imports raise significant concerns regarding human-induced environmental pressure. The COVID-19 pandemic resulted in a complete halting of tourism and extreme mobility restrictions, presenting a unique natural experiment to quantify how the abrupt reduction of human activity affects vegetation. This study provides the first joint assessment of how the tourism and agricultural sectors reacted in tandem to this mobility shock. Using satellite-based Leaf Area Index (LAI) data and a doubly robust difference-in-differences approach, we examine vegetation density shifts across both touristic and agricultural zones. Our findings reveal a significant 51% increase in vegetation density within tourism areas, specifically the fragile Bushes and Cacti zones, following the cessation of foot traffic. Concurrently, we document a 33% increase in LAI within agricultural zones relative to the counterfactual, signaling an intensification of local production. Further, these structural shifts in vegetation density persisted even as initial restrictions were eased. These quantitative results offer novel insights into the rapid responsiveness of island landscapes to changes in the human footprint, providing a data-driven foundation for resilient land-use policy.

## Introduction

The Galapagos Islands are globally renowned for their unique biodiversity and ecological sensitivity, while also being highly dependent on imported food and long relying on tourism as its dominant economic sector [1]. This combination creates a defining land-use challenge: how to balance conservation constraints with local food security and economic resilience in a small, protected island system [2].

Vegetation patterns in diverse ecosystems are substantially influenced by anthropogenic pressures, which often act as primary drivers of landscape modification. In island environments, human-induced disturbances, ranging from tourism-related infrastructure development to agricultural intensification, frequently alter natural successional trajectories, leading to fragmented landscapes and modified vegetation density [3].

Vegetation patterns in the Galapagos have been profoundly shaped by centuries of varying human pressure, characterized primarily by the introduction of invasive species and the conversion of land for agriculture [4]. More recently, the conversion of native forests and grasslands into agricultural land has transformed much of the non-protected humid zones into anthropogenic landscapes; for instance, native vegetation in the agricultural areas of Santa Cruz Island plummeted from 94% in 1961 to just 7% by 2018 [5]. While the growth of the tourism sector from the 1990s onward initially reduced direct pressure on some agricultural resources by offering alternative livelihoods, the underlying threat of human-mediated biological invasion remains the primary driver of modern vegetation dynamics across the archipelago [5].

In 2020, the COVID-19 pandemic led to an abrupt halt in tourism and external movement, including food imports, sharply reducing human activity in fragile habitats [6,7]. Touristic zones that typically experience heavy foot traffic experienced a sharp reduction in human disturbance, while disruptions to imports and tourism-related employment prompted a reorientation of local economic activity. In response, many residents turned to agriculture, cultivating previously underutilized land within existing designated agricultural zones to meet local demand [8].

These conditions created a natural experiment to study how reductions in external human pressure affect vegetation dynamics and land use. We examine changes in vegetation density using the Leaf Area Index (LAI), capturing both reduced disturbance in touristic zones and increased land-use intensity in agricultural areas [9]. In order to capture this vegetation response, we apply a doubly robust (DR) Difference-in-Difference (DiD) approach. This is combined with a sensitivity analysis and machine learning methods, to ensure the robustness of our results.

Existing studies have not directly quantified how restricting external human access (through reduced tourism and imports) affects vegetation dynamics and agricultural responses in protected island systems. Crucially, research has typically treated tourism-related ecological disturbance and agricultural land-use as separate phenomena; there is currently no joint assessment of how these two sectors reacted in tandem to the unique pressures of the COVID-19 pandemic. This gap is particularly relevant given that such restrictions are central to conservation policy but remain empirically under-evaluated. We address this gap by leveraging the COVID-19 shock as a natural experiment, providing the first quantitative evidence from a highly protected small-island context using satellite-derived vegetation indices.

This study provides three primary contributions. First, we offer empirical documentation of vegetation resurgence during a period of diminished human activity, enriching the ecological economics literature on human-nature trade-offs [10,11]. Second, while previous studies on island resilience have relied on qualitative and case-based approaches [8,12,13], we utilize satellite-derived LAI to provide a spatially detailed, quantitative analysis of land-use shifts. Finally, our findings establish the first joint assessment of how tourism-dependent and import-reliant systems can simultaneously experience ecological recovery and agricultural restructuring, providing a novel framework for evaluating conservation policy during global crises.

## COVID-19 and public policies in the Galapagos

Due to its importance to tourism and biological research, Ecuador has implemented a control system for human activity in the Galapagos islands to preserve the natural habitats. These policies span from agricultural restrictions to migration control [14]. Due to the COVID-19 pandemic, the Emergency Operations Committee (“Comité de Operaciones de Emergencia”, COE) carried out a border closure that not only banned national and international flights but also eliminated the ability to import resources from the continent [15]. Particularly, on March 16th of 2020, the movement of people and goods in and out of the islands was banned [15]. The first guidelines for the movement of foreigners and transients in the archipelago were issued in June 2020 [16]. The zero touristic activity restriction was broken for the first time in October 2020, when the entry of foreigners for tourism was allowed with significant restrictions through the second plan of mobilization for foreigners and transients [17]. Thus, the modifications were guided by the updating of vaccines and mobility requirements until October 2022 [18]. When finally, all requirements to enter the archipelago were abolished.

This movement ban left the already food insecure islands in a state of crisis. As a response, the Ministry of Agriculture (“Ministerio de Agricultura y Ganaderia”, MAG) in a teamwork with the Galapagos Government (“Consejo de Gobierno de Regimen Especial de Galapagos”, CGREG) started to develop plans to alleviate the crisis. Further, in 2021, it was decided to provide agricultural aid kits consisting of equipment, motor-shovels, and fertilizers to help to promote food security on the islands [19].

While these strategies to promote food security on the islands were developed in the years following the start of the pandemic, the precise timing of implementation and the extent of farmer participation remain unclear. Available reports indicate that the agricultural aid kits—consisting of equipment, motor-shovels, and fertilizers—were first distributed in 2021 [19], well after the onset of the border closure in March 2020 and after our primary treatment period of interest (March–October 2020). This suggests that the observed immediate changes in vegetation and land use during the peak restriction period were unlikely to be driven by these programs. Nonetheless, we acknowledge that subsequent government support could have reinforced or prolonged some of the behavioral shifts we document, and therefore our estimates for the post-2020 period may partially reflect the combined effect of the tourism/import shock and later agricultural aid.

## Data and methods

### Data

To analyze the impacts of the mobility restrictions, we work with data from different sources. Foliage density is captured through the leaf area index (LAI) collections at 300m dekad from the Copernicus Land Monitoring Service from March 2014 to February 2024. Likewise, precipitation data was obtained through the Climate Hazards Group InfraRed Precipitation with Station data (CHIRPS) [20] collection at UC Santa Barbara at a resolution of 300m dekad during the same period. Monthly average temperature records were obtained through the Copernicus Climate Change Service at a resolution of 0.5° for the same period. Additionally, the altitude of the islands was established using the Copernicus Global Digital Elevation Model 30m resolution.

To homogenize the panel series, we worked with a mesh of hexagonal cells with dimensions of 1 km x 1 km that overlap the vegetation cover map. Monthly averages were made for the LAI, precipitation, temperature and altitude. The use of a 1 km grid allows for consistent definition of treatment and control groups across space. At finer spatial resolutions (e.g., 300m or 500m), changes in grid boundaries can reassign cells across treatment and control groups, introducing potential contamination. Defining treatment and control areas at the 1 km level ensures stable group assignment across specifications.

Since we aim to study the effect of mobility restrictions on vegetation density, we focus our analysis on islands with both vegetation diversity and human activity. To define vegetation diversity, we use the land cover map from MapBiomas Ecuador 2022 [21], which has a resolution of 30m x 30m pixel size and contains over 30 types of land cover, 7 of which are identified in the Galapagos Islands (See Table 1). With this information, we compute the zonal histogram within the grid-cell and then calculate the relative share of each land cover with respect to the total pixels count within the grid-cell. Then, a given island had to have no more than 60% of its vegetation classified as Rocky Outcrop (see Table 1 for a description of the land cover classifications) to be selected for use in this study. This restriction was chosen to include islands with a diversity of vegetation types.

**Table 1:** Description of land cover classifications.

Land Cover Classification (Vegetation Type by MapBiomas in Galapagos)	*Description*
*Forest*	Arboreal vegetation located at an altitude between 400 and 700 meters (approximately). It can grow up to 20 meters high and 60 centimeters in circumference. May include transitional forest and invasive species.
*Open Forest*	Varied vegetation between short, open, thorny and cactus forests. Located between 100m and 300m. Includes deciduous and evergreen forests and may include invasive species.
*Mangroves*	Areas of dense, evergreen vegetation cover dominated by mangrove species.
*Grassland*	Areas composed of herbaceous vegetation.
*Rocky Outcrop*	Vegetation composed mostly of rock, including ancient and recent lava.
*Bushes and Cacti*	Deciduous shrublands with abundant cactus vegetation.
*River, Lake or Ocean*	Superficial water body. Can be artificial or natural.

Table adapted from [22].

**Fig 1:**
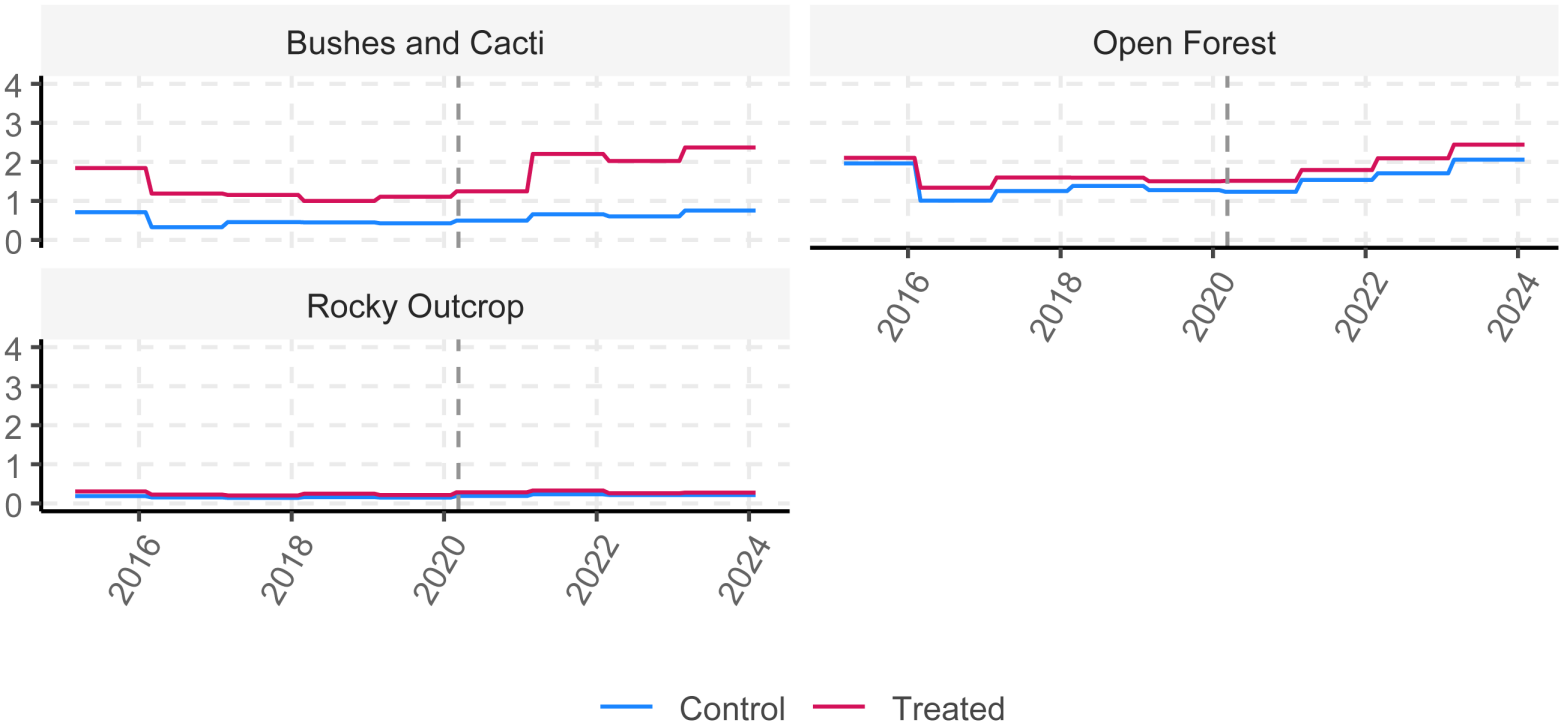
Evolution of the Leaf Area Index for tourism areas by vegetation type in the Galapagos Islands.

Among the islands that meet the criteria for study up to this point, we want to consider only those with human activity, for instance islands with permanent residents: Isabela, Santa Cruz, San Cristobal, and Floreana. We also add Santo Tomas Island since its size is similar to the populated islands and it has at least 10 touristic points on the island or within 1 km of its coast according to Charles Darwin Foundation’s tourist activities map [23]. Fig 1.A in Appendix A in S1 Appendices shows a map of the Galapagos and the selected islands. Note that blank grid cells represent areas that do not meet the criteria for either treatment or control groups and are therefore excluded from the analysis.

We split our analysis of human activity on the Galapagos Islands into touristic activities and agricultural activities. Importantly, we consider that the agricultural and touristic areas are mutually exclusive. Therefore, the grid-cells identified as agricultural areas (treated and control) are excluded from the touristic areas (treated and control), and vice versa. This classification is used to ensure clear separation between activity types and to avoid overlap that could confound the interpretation of vegetation responses. Also, notice that since we want to study the effect of the mobility restrictions on the LAI, it is important to identify a “control” region that is similar in all important dimensions to the treatment areas but are located outside documented tourism zones, agricultural land, and areas in proximity to roads or established trails within the selected islands. While it is not possible to rule out all human presence, these areas are defined as locations with no documented tourism or agricultural activity and are expected to have experienced minimal exposure to external human activity, thereby providing a plausible control group for assessing changes associated with the mobility restrictions. In the Galapagos, strict land-use regulations and enforcement by the National Park significantly limit human activity outside designated zones, which further supports the validity of these areas as low-exposure controls.

In order to prevent possible contamination of the treatment group with the control group, we excluded cells within 200 meters of the treatment areas from the analysis. In Appendix F, we further show that our results are robust across specifications that consider different potential contamination buffers areas and zones of influence. The following subsections outline how we determined which cells are classified as a tourism or agriculture areas and their respective control areas.

#### Tourism.

To determine the treated tourism grids, we use the following procedure. First, we identified locations (grid-cells) with touristic activities. We geo-locate these areas through the Charles Darwin Foundation’s tourist activities map [23]. Then, we combine this information to geo-referenced information (maps) of roads, routes, and walk-paths from the Governing Council of the Special Regime of Galapagos (*Consejo de Gobierno del Régimen Especial de Galápagos – CGREG*) (associated movement), to account for the movement of people to the tourism locations across Galapagos [24]. We assume that human movement associated with a point of tourism, roads and trails, could affect vegetation up to 500 meters around them.

Since we want to study the effect of the mobility restrictions on the LAI, it is important to identify a “control” region that is similar in all important dimensions to the treatment areas but was not affected by the mobility restrictions because it was never subject to external human activity in any reasonably known way. Hence, to guarantee the comparison between homogenous vegetations, the vegetation and land cover map from MapBiomas Ecuador 2022 [21] was used to characterize the hexagons of the grid-cell map. Therefore, for the tourism areas, we classify the cells by type of vegetation so that we have treatment and control groups corresponding to the same vegetation types (see Table 1). A given cell was classified into one of these groups if it has at least 80% of the total cell area covered by the respective vegetation type. Any cell that was not covered by one of the vegetation types by at least 80% was not considered. As tourist activities on the studied islands occur primarily in two main vegetation type areas: Open Forest, and Bushes and Cacti type areas, covering several cells subject to analysis, our study is limited to these vegetation type areas. In order to assess the robustness of our results, we also study the Rocky Outcrop as an area with low vegetation. The Forest vegetation type area covers relatively few cells in the touristic areas (less than 0.6%), and therefore it is excluded from the analysis. Hence, for the touristic treated areas corresponding to a type of vegetation, the control area corresponds to the same vegetation type, but without external human activity.

**Fig 2:**
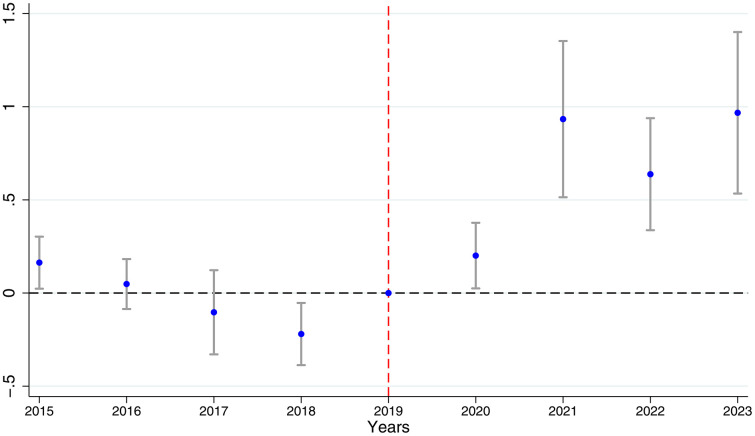
Effects on the LAI across touristic areas within the Bushes and Cacti vegetation type zones.

Figures Fig 2.A through Fig 6.A in Appendix A in S1 Appendices show the distribution of the grid cells between treatment and control for tourism and agricultural areas across the islands.

Table 2 shows the average, minimum, maximum, and standard deviation of the Leaf Area Index as derived from the data, temperature and rainfall for the different vegetation types across the agricultural and touristic areas between 2015 and 2024. Average LAI levels are higher in agricultural areas, relative to the rest of vegetation from the tourism areas. Further, it is important to note that the relatively low mean values are consistent with the vegetation structure of the Galapagos, which is dominated by shrublands, cacti, and open forest systems with lower canopy density.

**Table 2:** Summary statistics.

Variables	Mean	Min	Max	Standard Deviation	Observations
**Agriculture Areas**					
Leaf Area Index	2.133	0.000	7.000	0.414	19,230
Rainfall	2.838	0.066	49.220	0.745	19,233
Temperature	23.413	18.220	27.545	1.010	19,233
**Tourism Areas**					
**Bushes and Cacti**					
Leaf Area Index	0.649	0.000	6.017	0.189	1,791
Rainfall	2.669	0.000	45.676	0.613	1,935
Temperature	23.690	18.768	27.545	1.022	1,935
**Open Forest**					
Leaf Area Index	1.599	0.000	6.213	0.355	5,553
Rainfall	2.840	0.070	51.563	0.796	5,634
Temperature	23.441	18.220	27.545	1.015	5,634
**Rocky Outcrop**					
Leaf Area Index	0.183	0.000	5.833	0.033	22,869
Rainfall	2.868	0.000	46.742	0.756	23,382
Temperature	23.231	18.220	26.889	0.980	23,382

In summary, in tourism areas, we compare vegetation dynamics to similar areas located outside designated tourism zones over the full analysis period. Accordingly, the control group consists of grid cells located outside designated tourism areas, where access is restricted and human activity is expected to be minimal

#### Agriculture.

It is important to note that in the Galapagos Islands agriculture is limited to specific zones. Agricultural treatment cells were therefore determined by using the land use maps of the Galapagos as defined by the *Ministerio de Agricultura y Ganadería* (MAG).

A correct analysis of the effect of mobility restrictions on vegetation must compare treatment cells to a suitable control group. That is, in the control cells, agriculture was never allowed, and tourism was not known to be present. To be considered for the control group for agriculture, a non-treated cell must be in the same altitude range as the agricultural zones. Therefore, the elevation raster of the Copernicus Global Digital Elevation Model was used to identify the altitude range for the control group. Since the altitude data is available at higher resolution than the defined grid cells, we compute within grid average altitude across agricultural areas. The resulting range goes from 67 to 941 meters above sea level (masl). Additionally, since our outcome variable measures vegetation biophysical conditions, the control areas must be similar to the agricultural zones in that dimension. Comparing historical maps [25] and modern classifications of wild vegetation [26], we determined that the vegetation types Forest and Open Forest most closely approximates the agriculture zones, in terms of canopy and height of the vegetation. Further, our control group is made up of grids that contain at least 80% Forest or 80% Open Forest land and have no external human activity in the 5 islands selected for analysis.

In short, for the agricultural analysis, we compare the vegetation dynamics of the designated agricultural areas with the areas in which agriculture was never allowed (and were therefore not affected by the resultant shock from the restrictions of external activity). It is important to note here that the main channel in which the mobility restriction affected the agricultural zones is through the lack of imports and the necessity for local production.

### Methodology

To quantify the impact of reduced human activity on vegetation density in the Galapagos Islands, we exploit the natural experiment provided by the COVID-19 mobility restrictions initiated in March 2020. We utilize a panel of grid-cells across the archipelago, defining the treatment group as areas previously subject to human activity (touristic or agricultural) and the control group as similar areas never subject to such activity (see Appendix A for map distributions).

#### Difference-in-differences framework.

We employ a Difference-in-Differences (DiD) framework to estimate the average treatment effect on the treated (ATT). The baseline analysis uses a two-way fixed-effects (TWFE) regression to control for grid-cell invariant characteristics and common annual shocks. Since the mobility restrictions were implemented simultaneously across the islands, this design avoids the biases typically associated with staggered treatment settings.

#### Doubly robust estimators and covariates.

To ensure the validity of the parallel trends assumption, we implement doubly robust (DR) estimators following Callaway and Sant’Anna (2021) [27]. This approach combines regression adjustment (RA) and augmented inverse probability weighting (AIPW), providing consistent estimates even if one of the models is misspecified.

We control for key biophysical factors (precipitation, temperature, and altitude) that influence vegetation density. To incorporate these as dynamic variables, we extend the standard framework following Caetano and Callaway (2024) [28], using both within-grid-cell averages and changes from the base period to account for time-varying confounding factors.

#### Sensitivity analysis.

Following Rambachan and Roth (2023) [29], we evaluate the robustness of our results to potential violations of parallel trends. We use this test as a formal criterion to decide which results can be interpreted causally. Unlike traditional tests that rely on passing a pre-trend test, this framework allows us to quantify how much the parallel trends assumption would need to be violated to invalidate our conclusions. We apply Relative Magnitude (RM) restrictions, which bound potential post-treatment violations by a multiple of the largest observed pre-treatment deviation. Additionally, we use Second Derivative (SD) restrictions, which assume that any violation of parallel trends evolves smoothly over time, restricting the degree of non-linearity in the underlying trend differences. By identifying the breakdown frontier for our estimates, we provide a transparent basis for the causal interpretation of the impacts observed in both touristic and agricultural zones.

## Results

We divide our presentation of the results into the subsections of Tourism and Agriculture. In addition, we complement the results with a sensitivity analysis on the underlying assumption of the parallel trends. Furthermore, we run robustness estimations altering the contamination buffer areas. Finally, we run falsification tests to rule-out confounding effects that may affect the results (Appendix C).

### Tourism

We analyze the results across the three types of vegetation in the tourism areas: Bushes and Cacti, Open Forest, and we perform a falsification test analyzing Rocky Outcrop. The LAI for the tourism areas and its control follow the same trends prior to the start of the pandemic. However, Fig 1 shows that the evolution of the LAI for Bushes and Cacti vegetation (upper left panel) deviates from its control starting at the beginning of the pandemic and this difference appears to persist in the following periods. In contrast, Fig 1 shows that the Open Forest and Rocky outcrop areas follow similar trends after the mobility restriction (pandemic period).

Table 3 complements the graphical evidence and shows a strong positive effect of the mobility restrictions over the LAI increase of Bushes and Cacti vegetation type, in which the ATT estimates are robust across different specifications. Column (1) estimates a TWFE regression and finds that LAI increases in 0.69 columns (2) and (3) follows Callaway & Sant’Anna (2021) [27] doubly robust estimation approach and finds an effect of 0.702 and 0.685 of LAI increase, respectively. Columns (4) and (5) follows Caetano and Callaway (2024) [28] doubly robust estimation approach with dynamic covariates, finding a LAI increase of 0.678 and 0.589, respectively. Column (3) is our preferred and most conservative estimation approach, since this type of estimation is generally accepted (see [27]) relative to recent new alternative methodologies (see [28]).

**Table 3:** Effects of mobility restrictions on Leaf Area Index of Bushes and Cacti vegetation across touristic areas.

	Leaf Area Index				
	(1)	(2)	(3)	(4)	(5)
ATT	0.690***	0.702***	0.685***	0.678***	0.589***
	(0.149)	(0.127)	(0.146)	(0.147)	(0.148)
Weather controls	–	–	X	X	X
Altitude	–	–	X	X	X
Year fixed effects	X	–	–	–	–
Grid-cell fixed effects	X	–	–	–	–
Observations	1,791	1,791	1,791	1,791	1,576
Number of grid-cells	215	215	215	215	215

Difference-in-differences estimation using TWFE is in column (1). Callaway-Sant’Anna (2021) estimation approach is used in columns (2) and (3). Following Caetano and Callaway (2024), column (4) estimates the effects using within grid-cell average of the covariates, while column (5) controls for the change in the covariates from base-period and the level of covariates in the base-period. See text for details on specification and covariates. Standard errors are clustered at the grid-cell level and are shown in parentheses, significance levels at * p < .1, ** p < .05, *** p < .01.

We complement these results by estimating the dynamic effects in Fig 2, which shows an increase of LAI in the first post-treatment period, and stronger increases in the subsequent periods, relative to the pre-treatment periods. This result is explained by the growth of vegetation in touristic areas exposed to human activity and tourism after the mobility restrictions, even with the resumption of touristic activity. In contrast to the idea that touristic activity may affect the flora of any ecosystem, this evidence shows that the vegetation increased in these areas in the following post-treatment periods. We further explore this argument in Table 1.D in S1 Appendices showing Doubly Robust estimates with the collapsed panel data for the periods before, during and after the mobility restrictions. Column (2) of Table 1.D in S1 Appendices shows that the LAI of Bushes and Cacti vegetation increased by 0.775 after the mobility restrictions.

**Fig 3:**
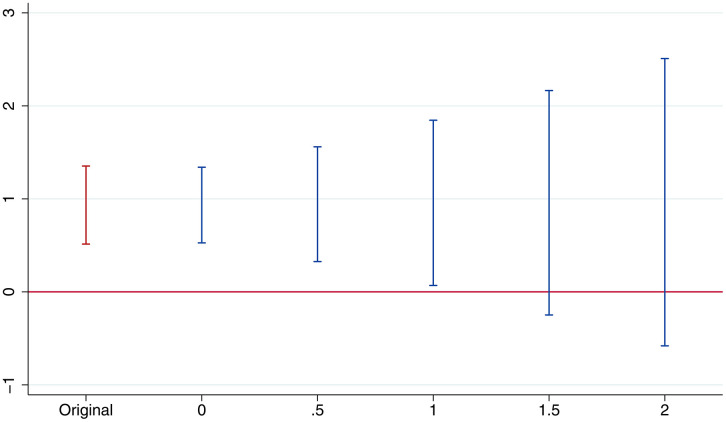
Sensitivity analysis of the second post-treatment period for the touristic areas within the Bushes and Cacti vegetation type zones: RM restrictions.

**Fig 4:**
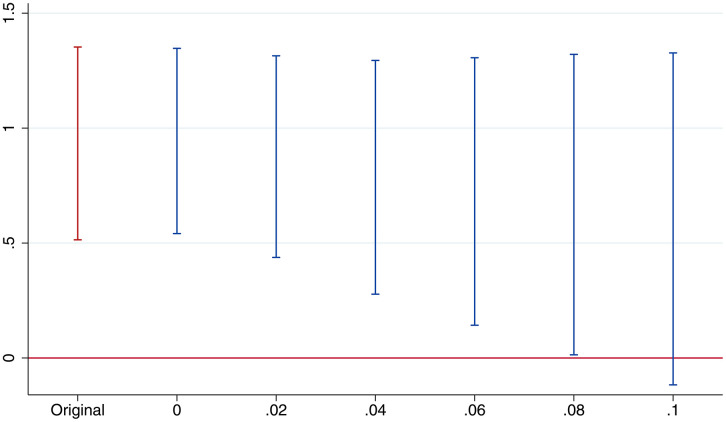
Sensitivity analysis of the second post-treatment period for the touristic areas within the Bushes and Cacti vegetation type zones: SD restrictions.

Figs 3 and 4 present the results for the sensitivity analysis of the ATT estimates over the second post-treatment period of Bushes and Cacti vegetation across touristic areas. The figure shows that the violation of the identifying assumption is small and that for our results to become insignificant, the violation of the identifying assumption needs to be at least the same size as the biggest violation in the pre-treatment period (that is $\overset{―}{M}=\text{1}$). In Fig 3, we apply the SD restrictions approach and find that the slope of the pre-trend can change up to about 8 percentage points for our results to become insignificant. Therefore, we can reject a zero effect unless the linear extrapolation across consecutive periods turns out to be more than 8 percentage points. We also test for sensitivity analysis for the ATT estimates over the average of the four post-treatment periods, and this is shown in the Appendix E, and the analysis show that, for our results to become insignificant, the violation of the identifying assumption needs to be at least half of the size as the biggest violation in the pre-treatment period (that is $\overset{―}{M}=\text{0}.\text{5}$). Also, if we apply the SD restrictions approach, we find that the slope of the pre-trend can change up to about 4 percentage points for our results to become insignificant.

In the case of the Open Forest vegetation type, the ATT estimates are shown in Table 1.B of Appendix B in S1 Appendices, and these are robust to the different specifications to estimate the ATT. However, there are concerns of pre-treatment differences across the Open Forest vegetation (see Fig 1.B in S1 Appendices). We next explore the sensitivity analysis of the ATT estimate for the second post-treatment period in figures Fig 1.B and 1.C (see Appendix B and C respectively in S1 Appendices). In contrast to the Bushes and Cacti vegetation, even though the ATT estimates suggest positive effects for Open Forest vegetation, the sensitivity analysis of the violation of identifying assumptions implies that the results do not hold. This is in line with the intuition that tourism and human activity mostly affect small-size vegetation, and it is less likely to affect the mid or big-size vegetation captured by satellite data. An additional consideration is that leaf formation of small crops and short-size vegetation such as Bushes and Cacti, that are located below mid- and big-size tree covers, cannot be clearly computed from Open Forest vegetation areas that are measured through satellites. Considering the above, we have decided to not interpret the results of the Open Forest vegetation type as causal and therefore, we focus our main analysis on the Bushes and Cacti vegetation areas.

Overall, there are positive effects on the LAI levels across touristic areas with Bushes and Cacti vegetation. The ATT estimates suggest, amidst the resumption of the touristic activity, an increase in the LAI of the Bushes and Cacti vegetation in the touristic areas with the presence of locals and visitors’ activity in the Galapagos. A potential explanation of these results is the local efforts to preserve the vegetation across touristic areas, in contrast to those areas without human activity with similar types of vegetation [30].

We also apply a supervised machine learning estimation [31,32] and find that the results are robust to these additional estimations. The methodological explanations and the results are shown graphically in Appendix G.

### Agriculture

**Fig 5:**
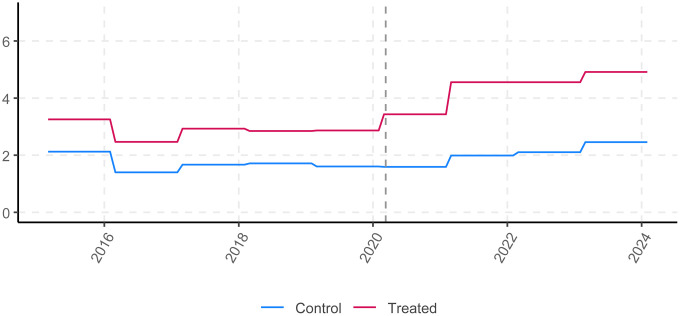
Evolution of the Leaf Area Index for agricultural areas in the Galapagos Islands.

Agricultural activities on the islands are restricted to certain areas, and therefore, we do not need to consider multiple vegetation types as in the case with tourism areas. The LAI for the agricultural areas and its control follow the same trends prior to the start of the pandemic. However, Fig 5 shows that the evolution of the LAI for agricultural areas in the Galapagos deviates from its control starting at the beginning of the pandemic and this difference persists in the following periods. Given strict land-use regulations in the Galápagos, the observed increases in LAI are consistent with intensified use or reactivation of underutilized land within existing agricultural zones and not that of large-scale horizontal expansion.

Table 4 complements the graphical evidence and shows a strong positive effect, in which the ATT estimates are robust across different specifications. Column (1) estimates a TWFE regression and finds that LAI increases in 1.081, columns (2) and (3) follow Callaway & Sant’Ana (2021) [27] doubly robust estimation approach and finds an effect of 1.017 and 1.066 of LAI increase, respectively. Columns (4) and (5) follow Caetano and Callaway (2024)’s [28] doubly robust estimation approach with dynamic covariates (see section 4), finding a LAI increase of 1.077 and 1.023, respectively. Similar to the case of tourism, we rely on Column (3) estimates to further analyze the results.

**Table 4:** Effects of mobility restrictions on the Leaf Area Index of vegetation across agricultural areas.

	Leaf Area Index				
	(1)	(2)	(3)	(4)	(5)
ATT	1.081***	1.017***	1.066***	1.077***	1.023***
	(0.0333)	(0.0364)	(0.0366)	(0.0371)	(0.0428)
Weather controls	–	–	X	X	X
Altitude	–	–	X	X	X
Year fixed effects	X	–	–	–	–
Grid-cell fixed effects	X	–	–	–	–
Observations	19,230	19,230	19,230	19,230	19,230
Number of grid-cells	2,137	2,137	2,137	2,137	2,137

Difference-in-differences estimation using TWFE is in column (1) with Callaway-Sant’Anna (2021) estimation approach in columns (2) and (3). Following Caetano and Callaway (2024), column (4) estimates the effects using within grid-cell average of the covariates, while column (5) controls for the change in the covariates from base-period and the level of covariates in the base-period. See text for details on specification and covariates. Standard errors are clustered at the grid-cell level and are shown in parentheses, significance levels at * p<.1, ** p<.05, *** p<.01.

**Fig 6:**
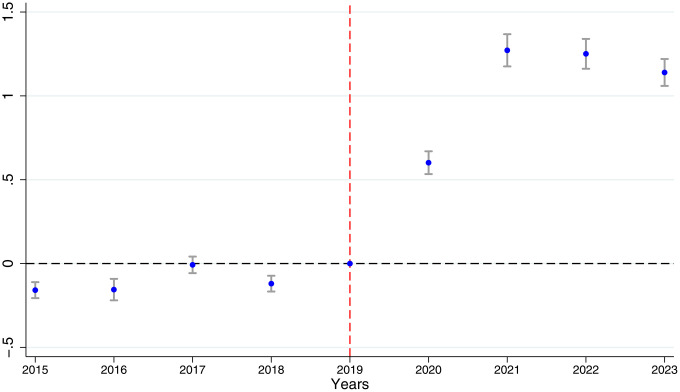
Effects on the LAI across agricultural areas.

We complement the previous results by estimating dynamic effects in Fig 6, which shows a relatively small effect in the first post-treatment period, that becomes larger in the subsequent periods. This result is explained by the agricultural cycle following the seeding and growing stages which requires time to generate leaf formation of vegetation across agricultural areas. We further explore this argument in Table 1.D (see Appendix D in S1 Appendices) showing the Doubly Robust estimates with the collapsed panel data for the periods before, during, and after the mobility restrictions. Column (1) of Table 1.D in S1 Appendices shows that the LAI increased by 0.457 during the mobility restrictions, and it increases by 1.265 after the restrictions were lifted. This result implies that the locals’ actions taken during pandemic to seed and grow agricultural products implied an increase in vegetation index of these areas after several months.

**Fig 7:**
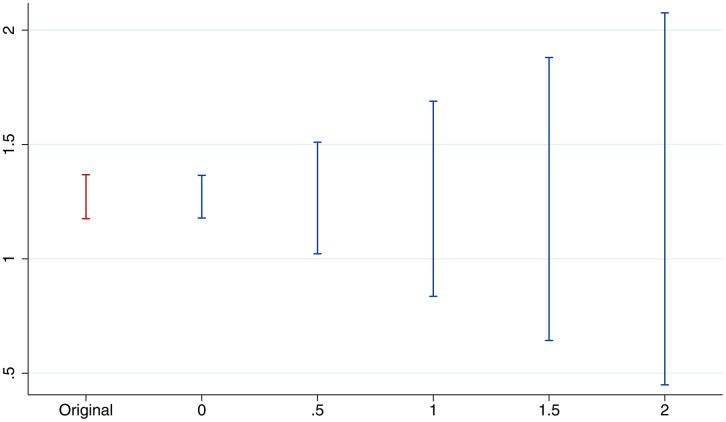
Sensitivity analysis of the second post-treatment period for the agricultural areas: SD restrictions.

**Fig 8:**
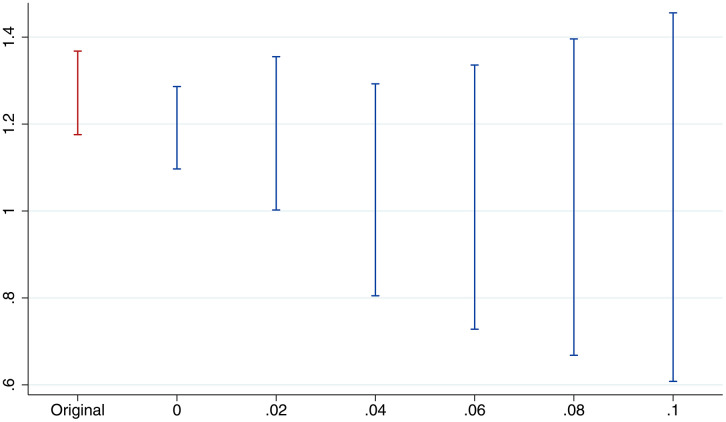
Sensitivity analysis of the second post-treatment period for the agricultural areas: RM restrictions.

When applying the event-study approach, the estimates before treatment are consistently smaller in absolute value than those after treatment, however, some of the former estimates are statistically different from zero (see Fig 7). To tackle potential concerns on the violation of the parallel trends assumptions that may affect the ATT estimates, we follow the method proposed by Rambachan and Roth (2023) [29]. We run the sensitivity analysis over the ATT estimates of the second post-treatment period. The results are presented graphically in Figs 6 and 8. Both figures show that the violation of the identifying assumption is small and does not imply a bias big enough to produce insignificant results. For instance, in Fig 8, we can see that for our results to become insignificant, the violation of the identifying assumption needs to be more than twice as big as the biggest violation in the pre-treatment period (that is $\overset{―}{M}=\text{2}$). Moreover, if we add SD restrictions (see Fig 7), the sensitivity analysis shows that the slope of the pre-trend can change up to values above the 10 percentage points for the ATT estimates to become insignificant. We also test for sensitivity analysis for the ATT estimates over the average of the four post-treatment periods (see Appendix E), in which the results draw qualitatively similar conclusions of the sensitivity of the results after taking into account the potential violation of parallel trends assumption.

We also apply a supervised machine learning estimation to flexibly control for covariates and we find that our results are robust to this additional set of estimations. The results are shown in Appendix G.

Overall, the results are robust across several specifications, and we show strong evidence that mobility restrictions brought significant increases in agricultural vegetation.

### Robustness checks

To assess the robustness of our main results, that is, the LAI in the agricultural zones and in the Bushes and Cacti vegetation type areas for the tourism zones, we perform additional estimations as follows.

#### Robustness check of temporal aggregation.

To assess whether temporal aggregation affects our results, we replicate the main difference-in-differences specification for the agricultural areas and for the Bushes and Cacti vegetation type in the tourism areas using monthly data rather than annual aggregates. The results, presented in Table 1.H of Appendix H in S1 Appendices, are more significant, but consistent, with our primary findings. This suggests that the estimated effects are not driven by temporal aggregation and that intra-annual variation does not materially alter the main conclusions.

#### Robustness check of buffer around treated areas and alternative land-use classification thresholds.

We assess the sensitivity of our results to the choice of contamination buffer by estimating the regression using alternative buffer distances (200m, 400m, 600m, and 1000m) around the treated areas. The results, reported in Appendix F, remain consistent across specifications.

Results are also robust to alternative land-use classification thresholds used to assign grid cells to treatment groups, indicating that the findings are not driven by the inclusion of mixed land-use cells (see Appendix I).

#### Robustness check of grid cell size.

To assess robustness to the size of the grid cells, we re-estimate the models using hexagonal grid cells of 500m and squared grid cells at 300m while maintaining these group definitions and find that the results remain consistent (see Appendix J).

#### Robustness check of tourism area identification.

We test the sensitivity of our findings by employing alternative radii to define tourism-treated areas, ranging from 300m to 1000m around tourism-related points of interest. The results remain consistent across these varying distances, indicating that the observed effects are robust and not contingent upon the initial 500m distance assumption. Detailed results for these alternative specifications are documented in Appendix K.

### Falsification and sensitivity analysis

We perform a falsification analysis with the Rocky Outcrop vegetation type following the intuition that Rocky Outcrop vegetation type is not likely to show damage from human activity due to the physical characteristics of the vegetation. The results are exhibited in Figs 1.B, 2.B and 3.B in S1 Appendices, which confirms the null effect of mobility restrictions over the formation of vegetation across this area. Therefore, an analysis of their results does not lend itself to useful interpretation. This is also suggestive evidence that our results of vegetation across the agriculture areas and the Bushes and Cacti vegetation across touristic areas, are not driven by general climate or time factors that affect LAI evolution in these areas. Therefore, we can rule out other confounding factors across vegetation in Agricultural areas and the Bushes and Cacti vegetation in touristic areas.

## Discussion and conclusions

Our findings demonstrate that the exogenous shock of the COVID-19 pandemic served as a catalyst for a dual transition in the Galapagos: a significant increase in vegetation density within tourism zones and apparent intensified production in agricultural zones. Although LAI cannot distinguish between endemic, invasive, and cultivated vegetation, our results are consistent with increases in vegetation density associated with reduced human pressure, though they do not provide direct evidence of broader ecological recovery. This use of LAI allows us to examine changes in vegetation density across both tourism and agricultural zones, capturing patterns consistent with reduced disturbance in some areas and increased agricultural activity in others, while recognizing its taxonomic limitations.

Specifically, in relevant tourism zones (Bushes and Cacti), LAI rose by approximately 51%, supporting the view that even regulated tourism can degrade vegetation in sensitive areas. The persistence of these patterns may be consistent with longer-lasting changes in vegetation density following reductions in tourism activity. These findings may have implications for ongoing conservation policy discussions in the Galapagos, particularly in the context of recent increases in entry fees, though further evidence would be needed to assess broader ecological outcomes.

At the same time, our analysis of satellite-based LAI data shows a 33% increase in vegetation density within agricultural zones relative to the counterfactual, consistent with intensified local food production likely driven by reduced imports and temporary labor reallocation from tourism. The strict land-use regulations in the Galapagos and the observed increases in LAI further suggest a reactivation of underutilized land within existing agricultural zones rather than a horizontal expansion. This adaptive response appears to have been maintained beyond the immediate crisis period. Importantly, we find no evidence that agricultural production expanded outside designated agricultural zones, underscoring the potential to boost food security while preserving native ecosystems. These findings indicate that temporary reductions in tourism and imports are associated with increases in vegetation density and sustained agricultural activity, reflecting concurrent adjustments in ecological conditions and food systems.

We further validated our findings through a series of robustness checks, confirming that the results are insensitive to changes in temporal aggregation, the size of contamination buffers, utilized grid cell size, and varying tourism area identification. The consistency of these results across different spatial and temporal parameters strengthens the reliability of our estimates and provides a rigorous baseline for future longitudinal studies in the archipelago.

These results have direct implications for small-island policy. They highlight the importance of regulated family farms in buffering food systems during supply chain disruptions and reducing the risks of invasive species introductions from imports. They also illustrate the potential of strategically timed tourism restrictions to preserve fragile vegetation. Beyond provisioning services such as food, these dynamics also affect regulating and cultural ecosystem services that underpin community well-being in the Galapagos. Future research should examine shifts in vegetation composition and assess effects on endemic fauna, but our findings offer encouraging evidence that targeted policies can promote both food system resilience and environmental preservation in ecologically significant island contexts.

Our study contributes to the ecological economics literature by providing unique empirical evidence of vegetation regeneration and potential ecosystem recovery under conditions of reduced human activity (e.g., [10,11]). By linking vegetation dynamics to both conservation outcomes and livelihood resilience, we highlight the interdependence of ecological integrity and human well-being in small-island systems [33–34]. In this sense, the paper adds to broader debates on conservation–development trade-offs [35–36], ecosystem resilience [37–38], and the design of policies that respect ecological limits while supporting food security [39].

This paper also contributes to the growing literature on adaptation and food security under crisis conditions [40–42] by leveraging a natural experiment in the import-dependent and ecologically preserved Galapagos Islands. Unlike other tropical regions where the pandemic was associated with higher rates of deforestation due to reduced monitoring and economic need [43], the Galapagos experienced a distinct combination of vegetational resurgence and forced food system adaptation. Whereas previous studies have relied on yield data or surveys to evaluate resilience [44–45], our analysis employs satellite-based vegetation data to provide a novel, spatially rich perspective in a data-scarce context [45–46].

To our knowledge, this is the first empirical study to show that satellite-based vegetation indices can be used to jointly examine changes in vegetation density and food production dynamics in a protected small-island system. In doing so, this study contributes to the literature on conservation–food security trade-offs by providing evidence that these processes can evolve concurrently under changing economic conditions. Overall, the study provides evidence that land-use dynamics can evolve in ways that reflect both conservation constraints and local livelihood needs in protected island systems, a central challenge for land-use policy and planning in small, vulnerable regions.

## Supporting information

S1 Appendices Appendices. This document contains Appendix A – Appendix K.
(DOCX)
