# Supplementary material for: Welcome to paradise: Measuring vegetation responses to tourism and agricultural activity using Leaf Area Index in the Galapagos Islands
Source: PLoS One. 2026 Jun 10;21(6):e0344628. doi: 10.1371/journal.pone.0344628 (PMC13252761; doi:10.1371/journal.pone.0344628)
Supplement: S1 Appendices — This document contains Appendix A – Appendix K. (DOCX) [file pone.0344628.s001.docx]

**Appendix A – Maps**


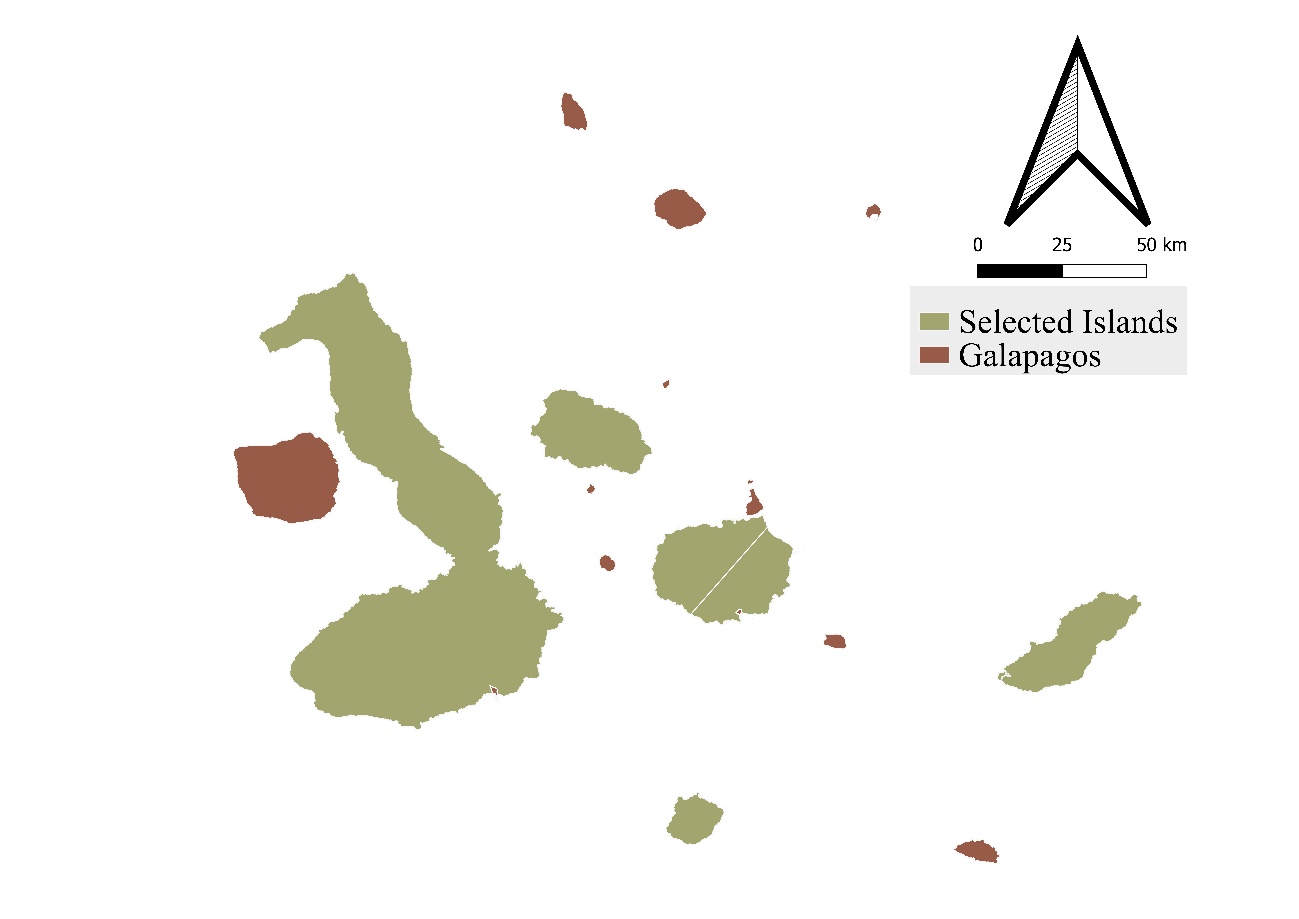


**Fig. A.1.** **Selected islands for the study.**


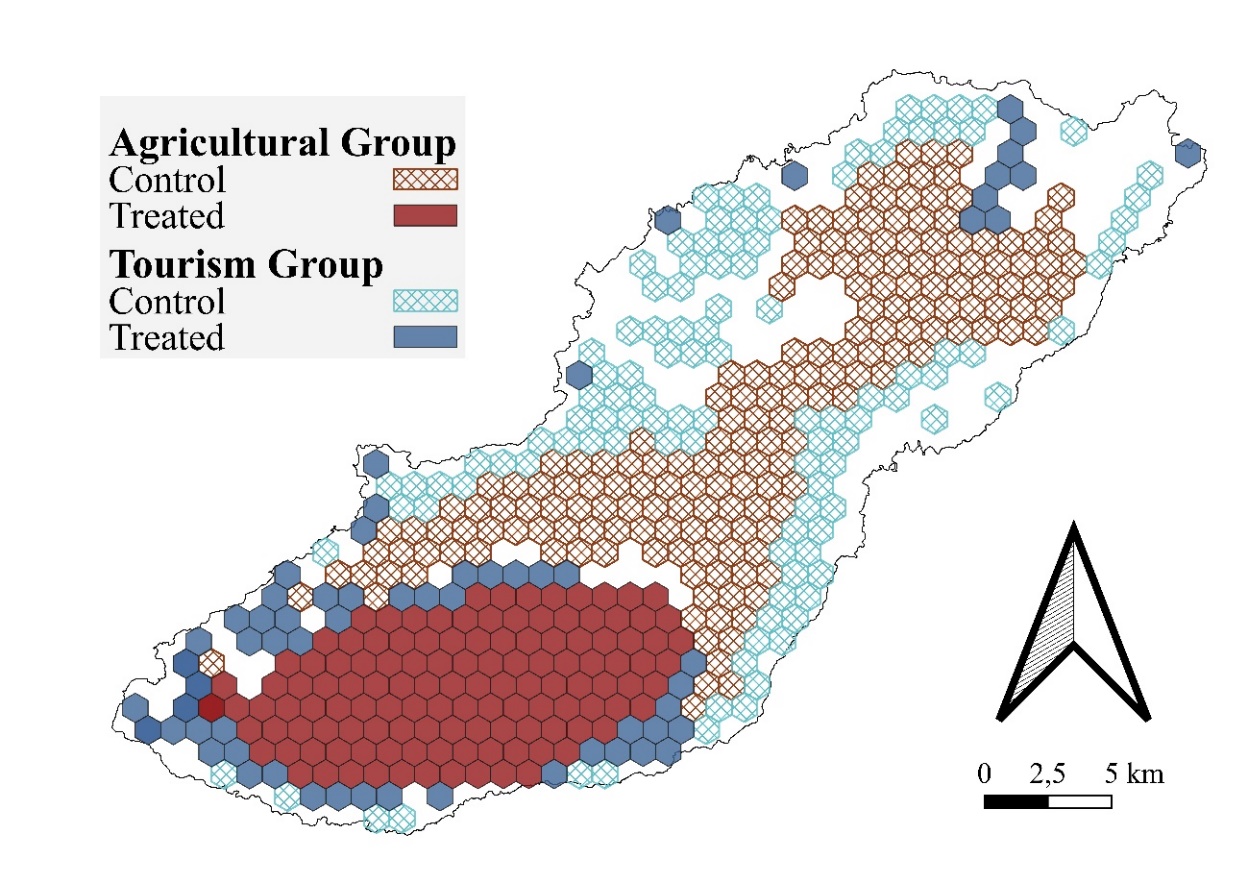


**Fig. A.2.** **Treatment and control cells for agriculture and tourism on San Cristóbal Island.**


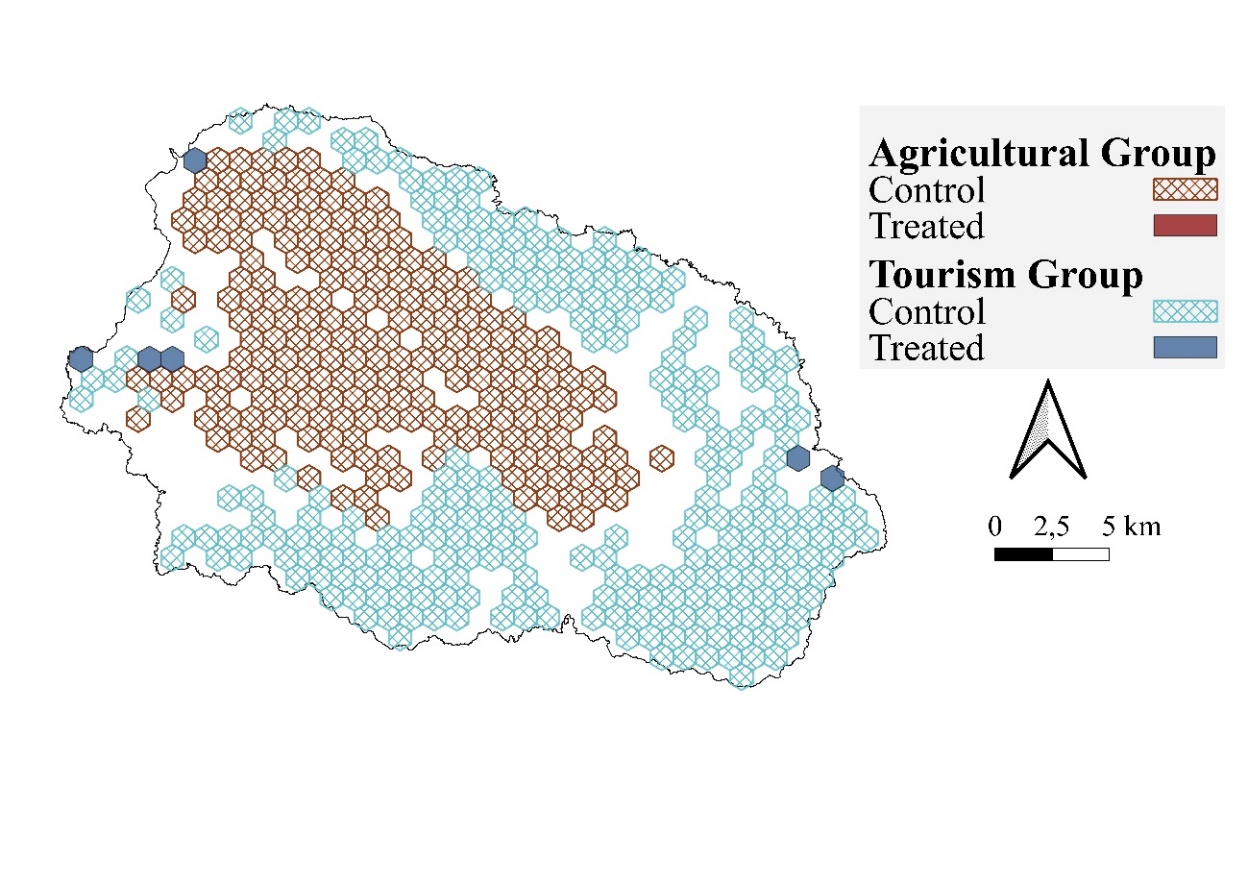


**Fig. A.3.** **Treatment and control cells for agriculture and tourism on Santo Tomás Island.**


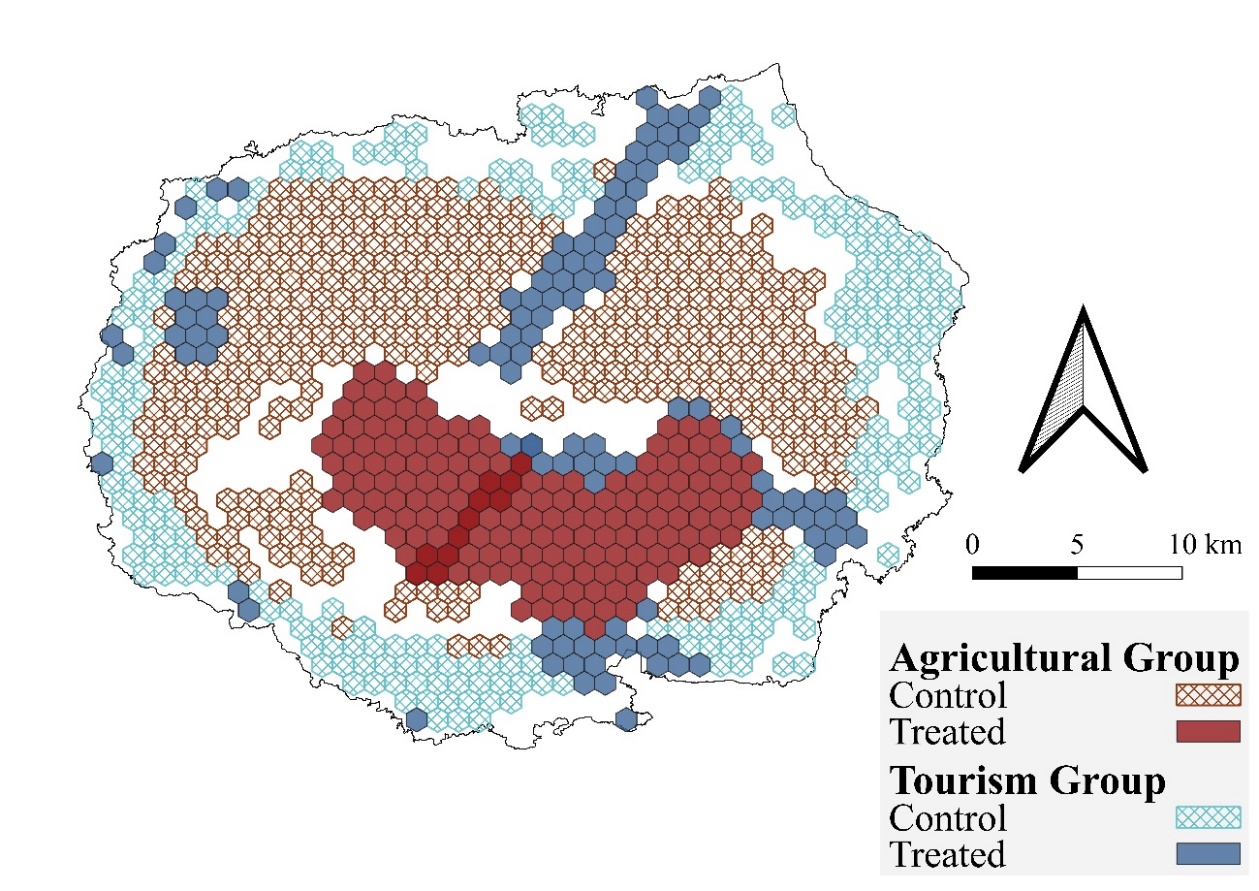


**Fig. A.4. Treatment and control cells for agriculture and tourism on Santa Cruz.**


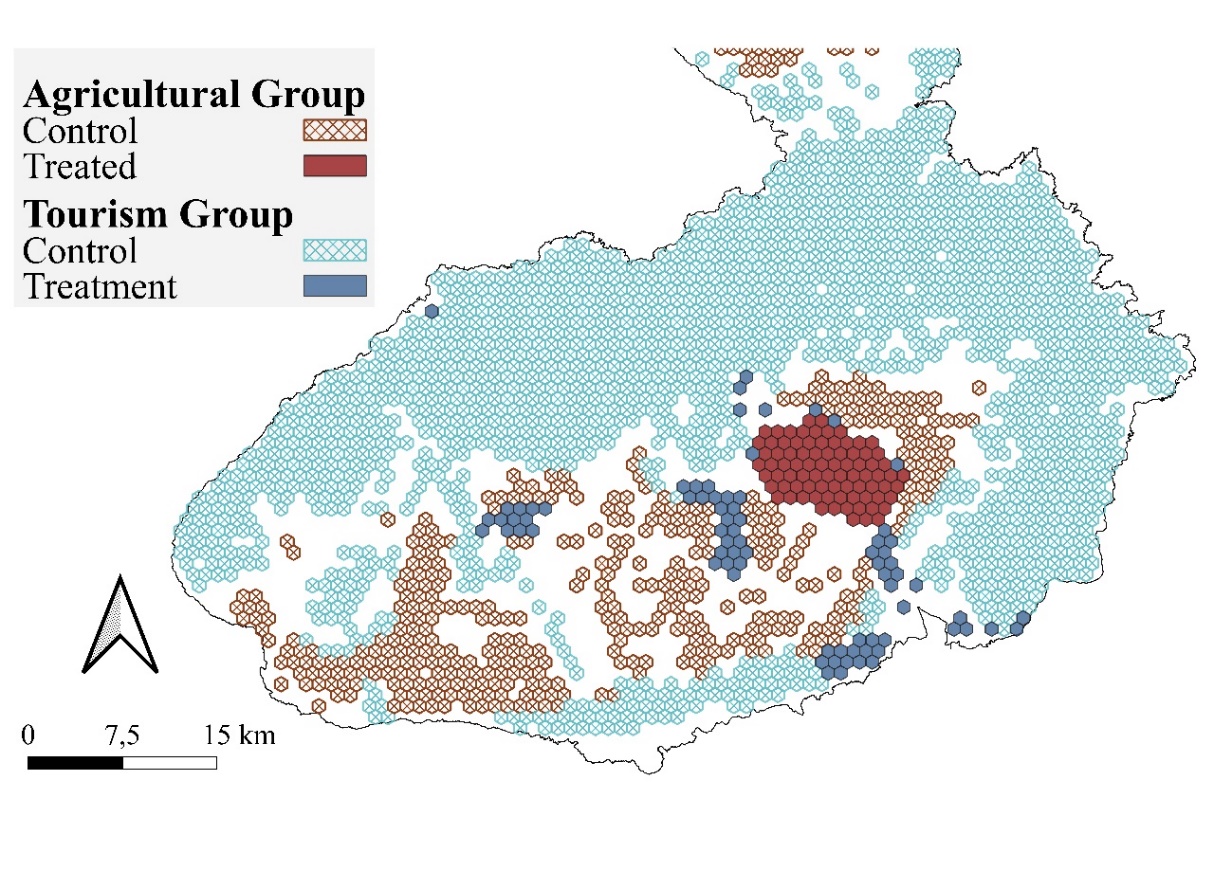


**Fig. A.5.** **Treatment and control cells for agriculture and tourism on the southern part of Isabela Island.**


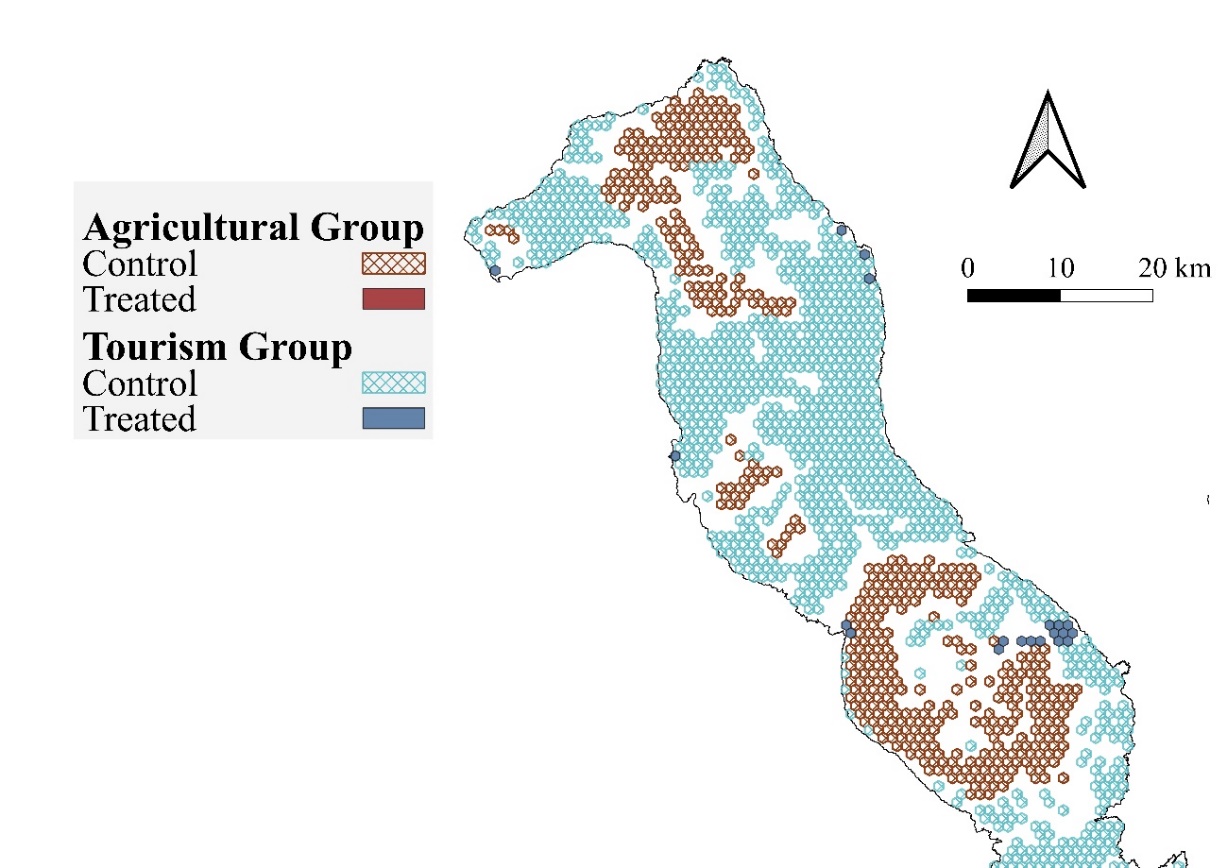


**Fig. A.6.** **Treatment and control cells for agriculture and tourism on the northern part of Isabela Island.**


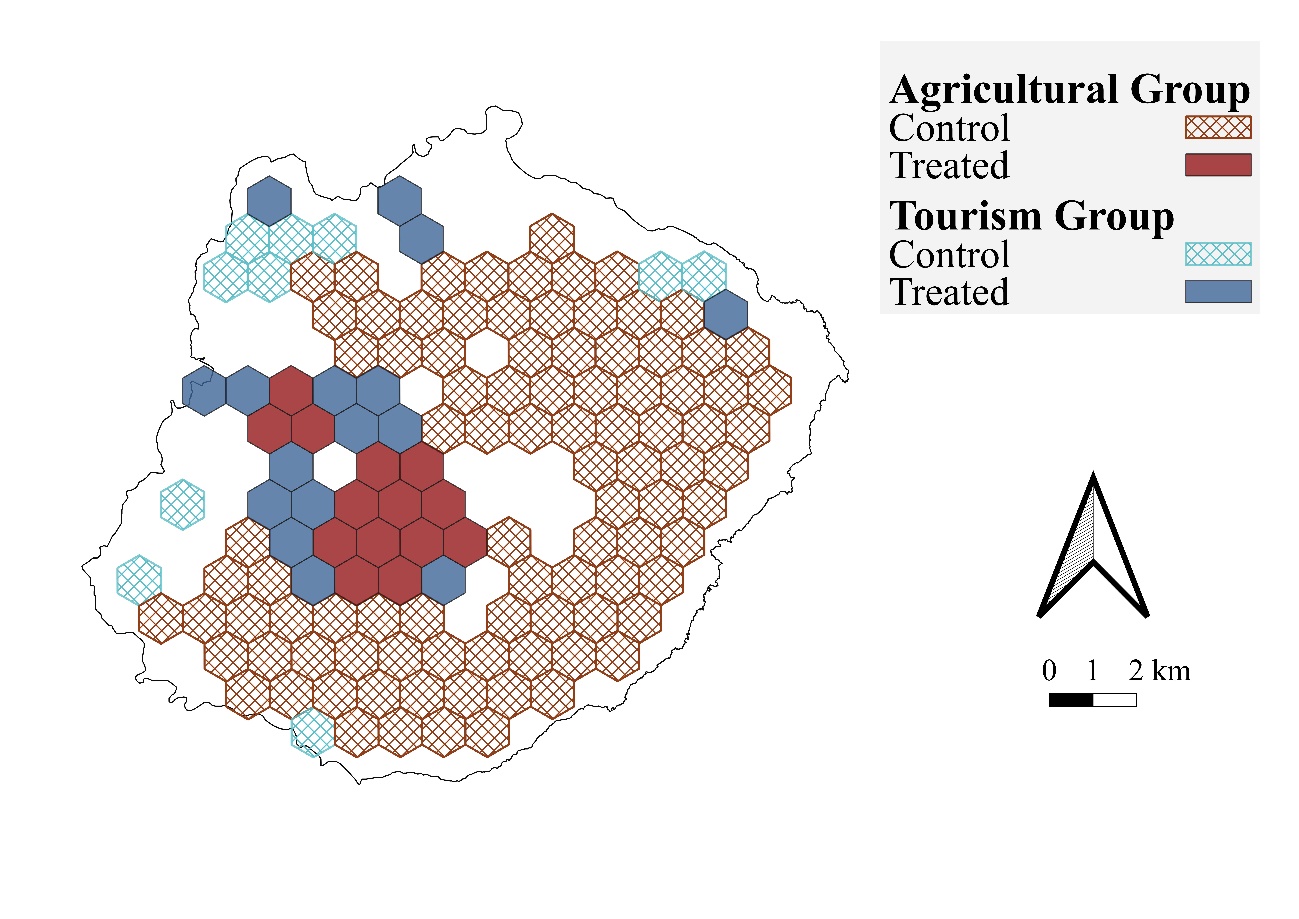


**Fig. A.7.** **Treatment and control cells for agriculture and tourism on the northern part of Floreana Island.**

**Appendix B – Analysis of Open Forest vegetation across touristic areas**

**Table. 1.B. Effects of mobility restrictions on Leaf Area Index of Open Forest vegetation across touristic areas.**

|  |  |  | Leaf Area Index | | | |  | |
| --- | --- | --- | --- | --- | --- | --- | --- | --- |
|  |  | (1) | (2) | (3) | (4) | (5) | |  |
|  |  |  |  |  |  |  | |  |
| ATT |  | 0.108** | 0.119** | 0.143*** | 0.157*** | 0.168*** | |  |
|  |  | (0.0383) | (0.0382) | (0.0382) | (0.0381) | (0.0362) | |  |
|  |  |  |  |  |  |  | |  |
| Weather controls |  | - | - | X | X | X | |  |
| Altitude |  | - | - | X | X | X | |  |
| Year fixed effects |  | X | - | - | - | - | |  |
| Grid-cell fixed effects |  | X | - | - | - | - | |  |
| Observations |  | 5,553 | 5,553 | 5,553 | 5,553 | 5,553 | |  |
| Number of grid-cells |  | 626 | 626 | 626 | 626 | 626 | |  |

Differences in Differences estimation using TWFE in column (1), Callaway-Sant’Anna (2021) estimation approach in columns (2) and (3). Following Caetano and Callaway (2024), column (4) estimates the effects using within grid-cell average of the covariates, while column (5) controls for the change in the covariates from base-period and the level of covariates in the base-period. Standard errors are clustered at the grid-cell level and are shown in parentheses, significance levels at * p<.1, ** p<.05, *** p<.01


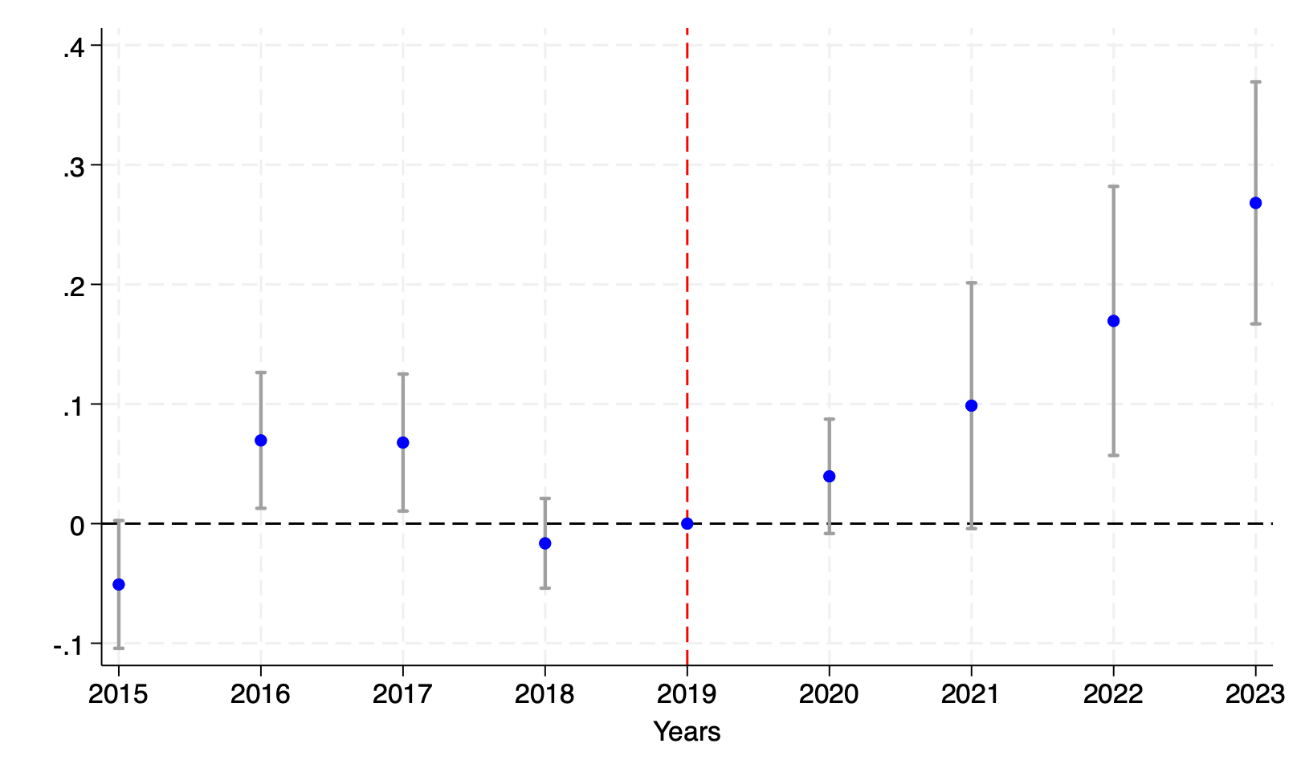


**Fig. 1.B**. **Effects on the LAI across touristic areas within the Open Forest vegetation type zones.**


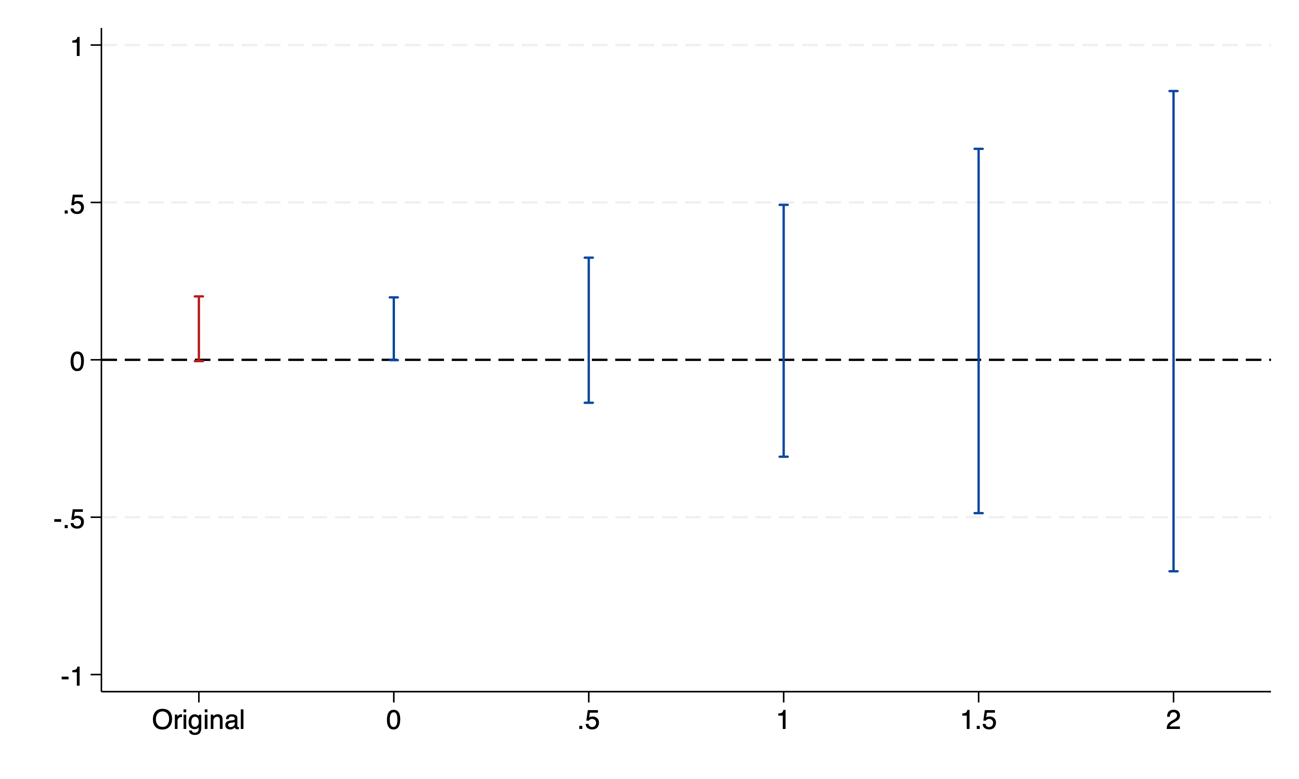


**Fig. 2.B.** **Sensitivity analysis of the second post-treatment period for the touristic areas within the Open Forest vegetation type zones: RM restrictions.**


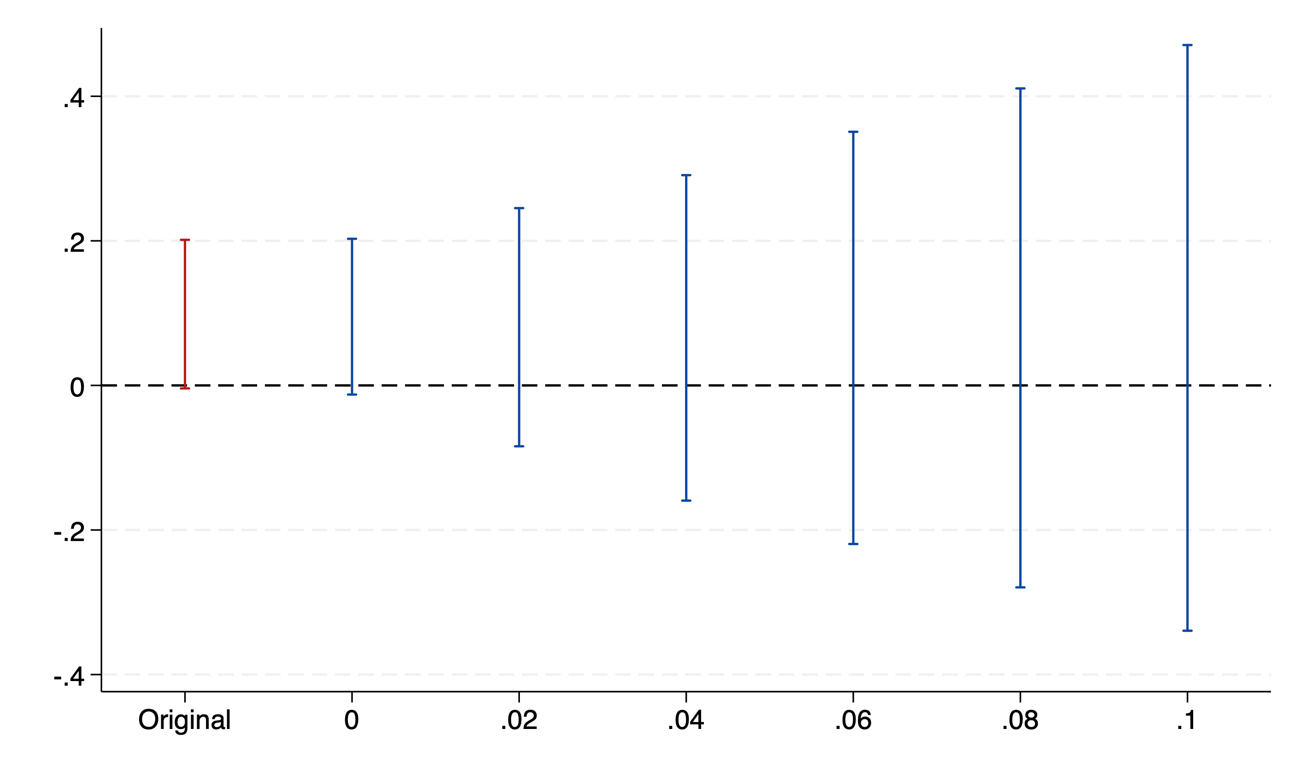


**Fig. 3.B.** **Sensitivity analysis of the second post-treatment period for the touristic areas within the Open Forest vegetation type zones: SD restrictions**

**Appendix C – Falsification analysis – Rocky Outcrop vegetation across touristic areas**

**Table. 1.C.** **Effects mobility restrictions on Leaf Area Index of Rocky Outcrop vegetation across touristic areas.**

|  |  |  | Leaf Area Index | | | |  | |
| --- | --- | --- | --- | --- | --- | --- | --- | --- |
|  |  | (1) | (2) | (3) | (4) | (5) | |  |
|  |  |  |  |  |  |  | |  |
| ATT |  | -0.0222 | -0.0515 | -0.00924 | -0.00792 | -0.0177 | |  |
|  |  | (0.0257) | (0.0234) | (0.0245) | (0.0240) | (0.0293) | |  |
|  |  |  |  |  |  |  | |  |
| Weather controls |  | - | - | X | X | X | |  |
| Altitude |  | - | - | X | X | X | |  |
| Year fixed effects |  | X | - | - | - | - | |  |
| Grid-cell fixed effects |  | X | - | - | - | - | |  |
| Observations |  | 22,869 | 22,869 | 22,869 | 22,869 | 22,869 | |  |
| Number of grid-cells |  | 2598 | 2598 | 2598 | 2598 | 2598 | |  |

Differences in Differences estimation using TWFE in column (1), Callaway-Sant’Anna (2021) estimation approach in columns (2) and (3). Following Caetano and Callaway (2024), column (4) estimates the effects using within grid-cell average of the covariates, while column (5) controls for the change in the covariates from base-period and the level of covariates in the base-period. Standard errors are clustered at the grid-cell level and are shown in parentheses, significance levels at * p<.1, ** p<.05, *** p<.01


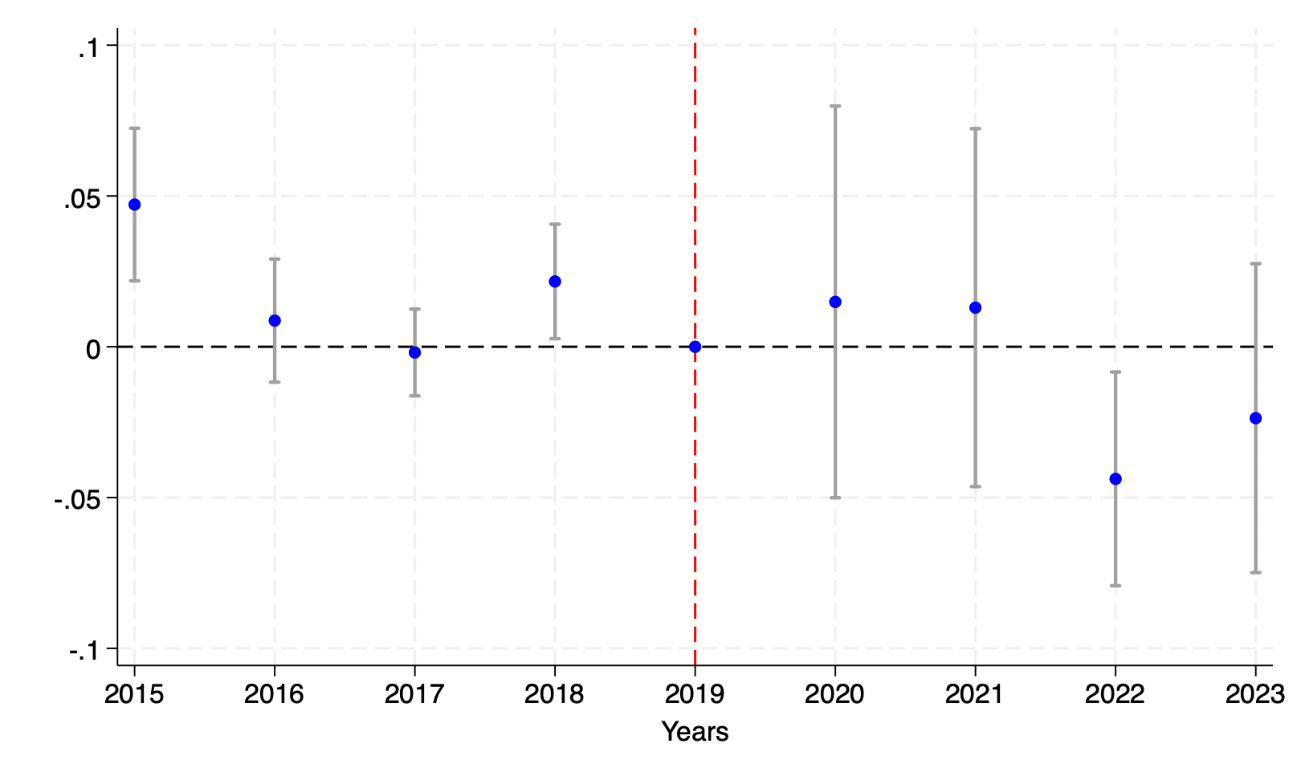


**Fig. 1.C.** **Effects on the LAI across touristic areas within the Rocky Outcrop vegetation type zones.**


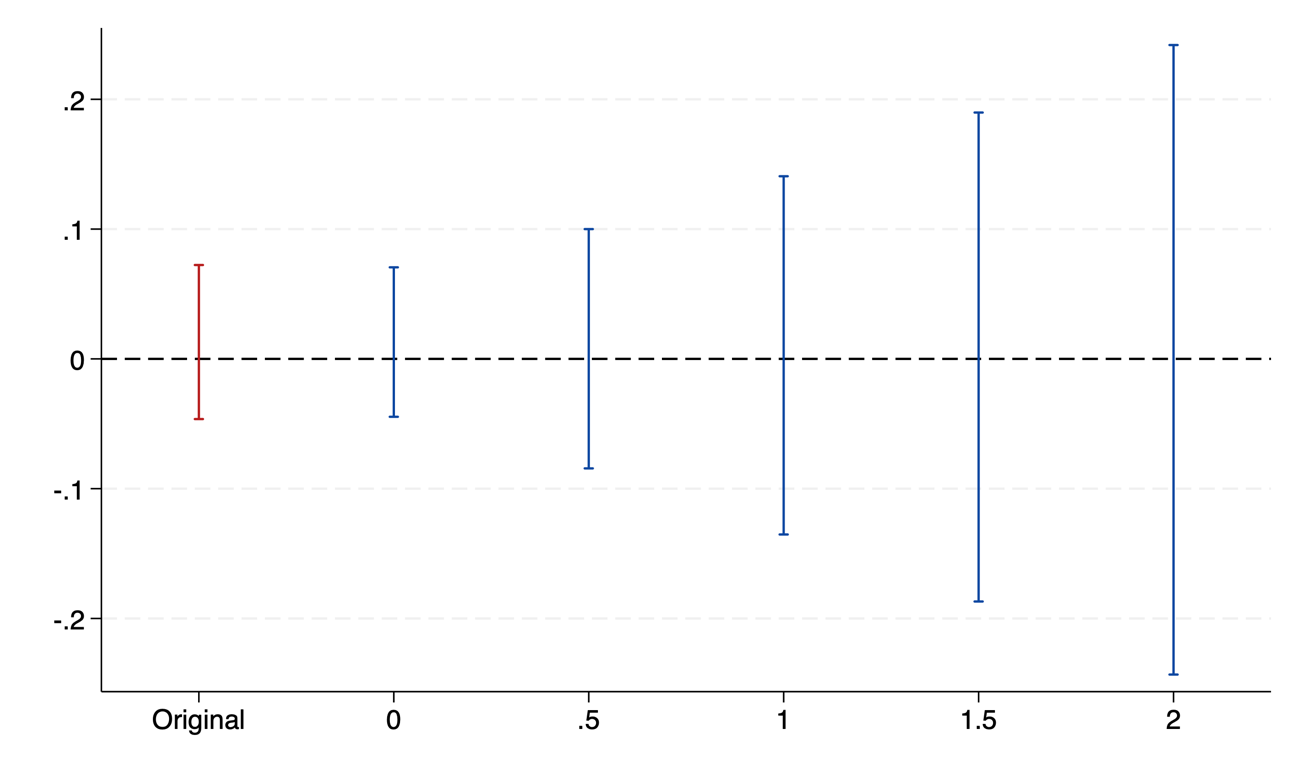


**Fig. 2.C.** **Sensitivity analysis of the second post-treatment period for the touristic areas within the Rocky Outcrop vegetation type zones: RM restrictions.**


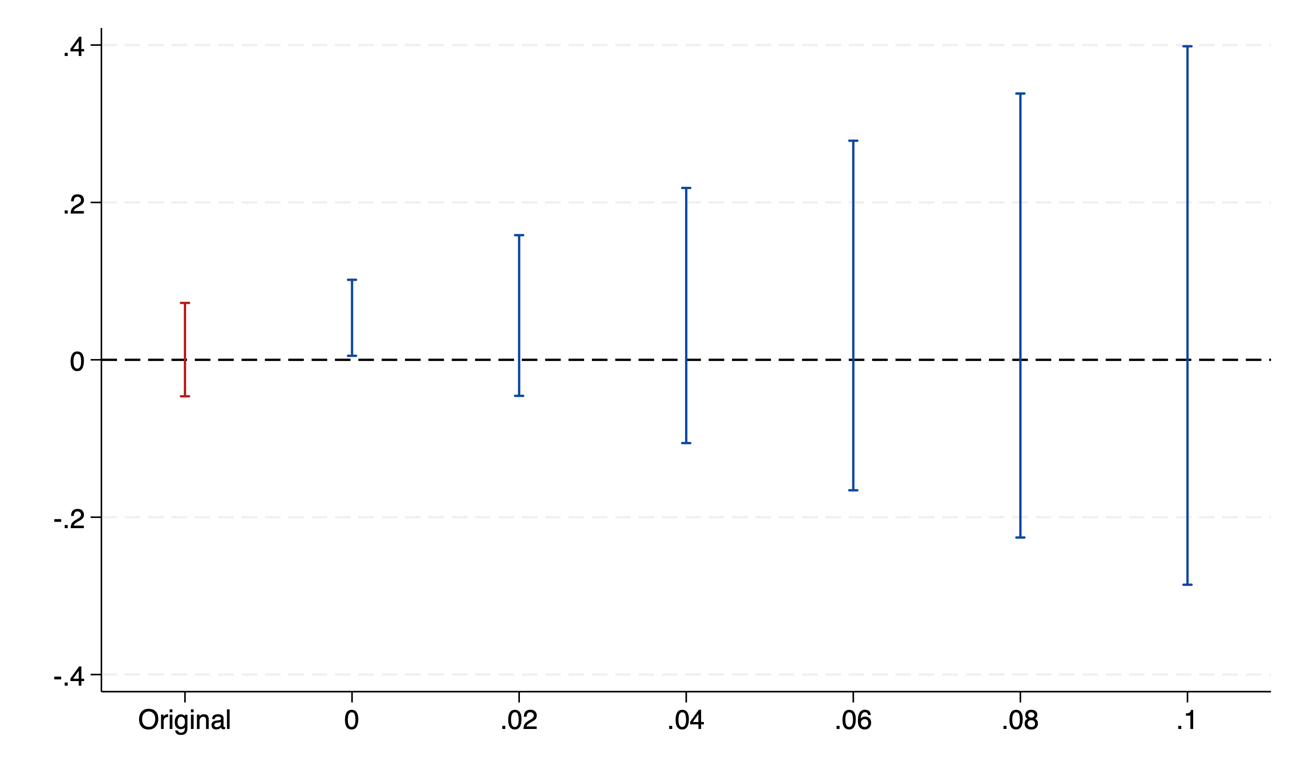


**Fig. 3.C.** **Sensitivity analysis of the second post-treatment period for the touristic areas within the Rocky Outcrop vegetation type zones: SD restrictions.**

**Appendix D -** **Effects mobility restrictions on Leaf Area Index across human activities - During and After Travel Restrictions.**

**Table. 1.D.** **Effects mobility restrictions on Leaf Area Index across human activities - During and After Travel Restrictions.**

|  | | Leaf Area Index | | | | |
| --- | --- | --- | --- | --- | --- | --- |
|  | | | Agriculture | Tourism | | |
|  |  | Bushes and Cacti | Open Forest | Rocky Outcrop |  |  |
| During mobility Restrictions | | | 0.457^***^ | 0.0594 | 0.0589^*^ | -0.0006 |
|  | | | (0.0352) | (0.120) | (0.0290) | (0.0342) |
|  | | |  |  |  |  |
| After mobility Restrictions | | | 1.265^***^ | 0.789^***^ | 0.192^***^ | -0.0254 |
|  | | | (0.0360) | (0.174) | (0.0444) | (0.0253) |
|  | | |  |  |  |  |
| Weather controls | | | X | X | X | X |
| Altitude | | | X | X | X | X |
| Observations | | | 6,410 | 597 | 1,851 | 7,623 |

Differences in Differences estimation using Callaway-Sant’Anna (2021) approach. Standard errors are clustered at the grid-cell level and are shown in parentheses, significance levels at * p<.1, ** p<.05, *** p<.01

**Appendix E - Sensitivity analysis of touristic and agricultural areas on the average of the effect over the four post-treatment periods**


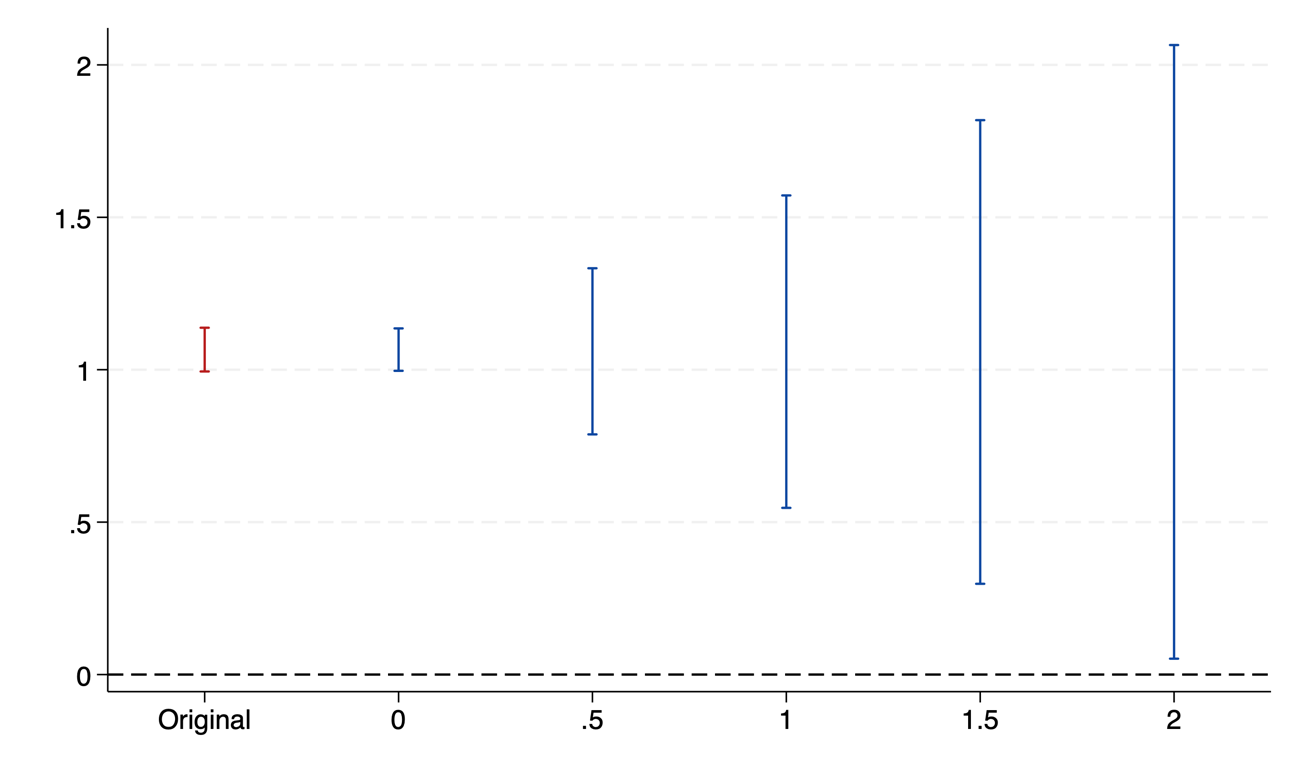


**Fig. 1.E.** **Sensitivity analysis of the average over the four post-treatment periods for the agricultural areas: RM restrictions.**


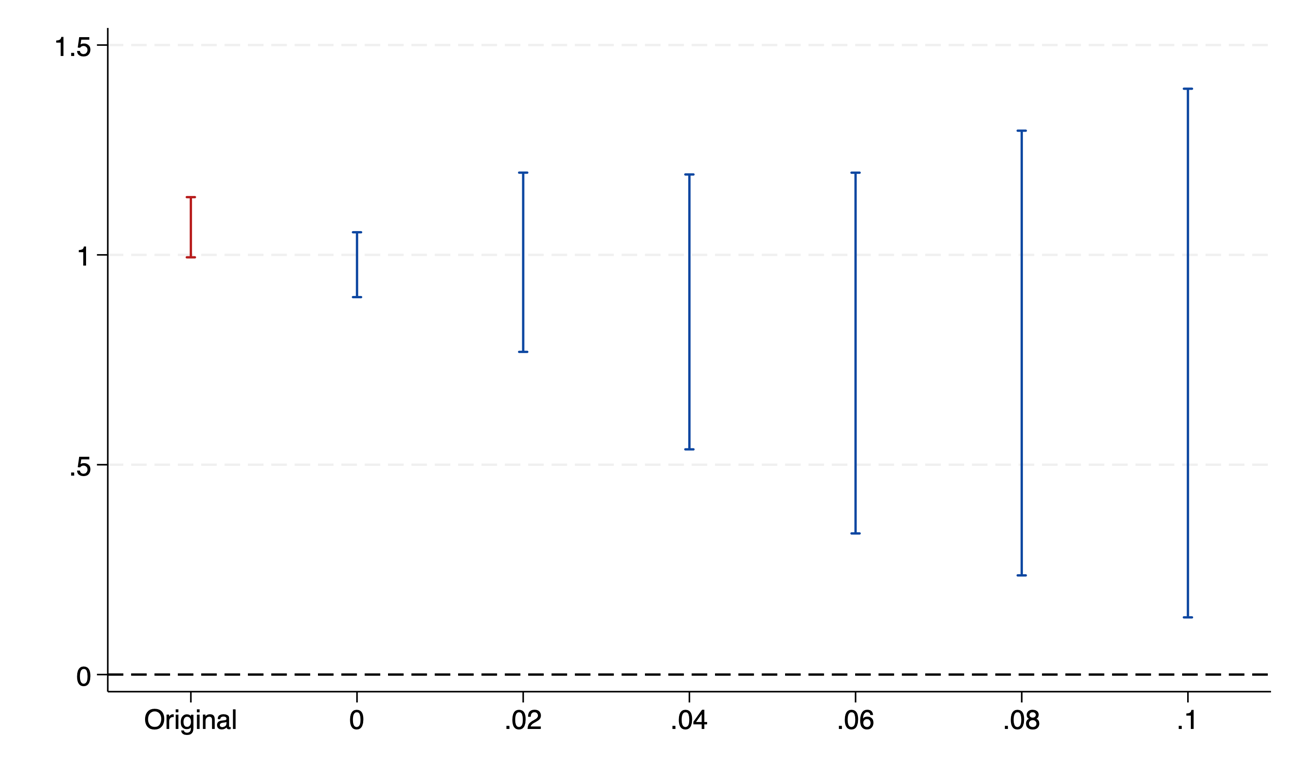


**Fig. 2.E.** **Sensitivity analysis of the average over the four post-treatment periods for the agricultural areas: SD restrictions.**


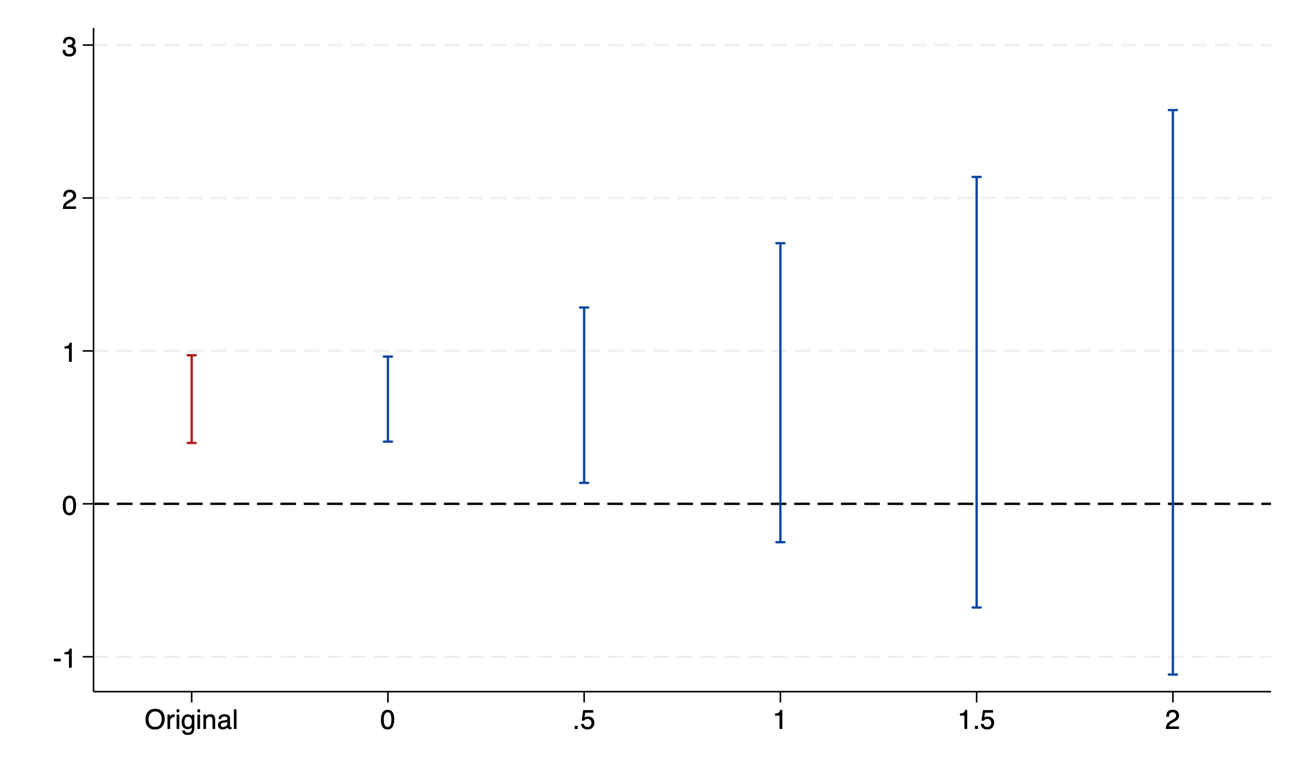


**Fig. 3.E.** **Sensitivity analysis of the average over the four post-treatment periods for the touristic areas within the Bushes and Cacti vegetation type zones: RM restrictions.**


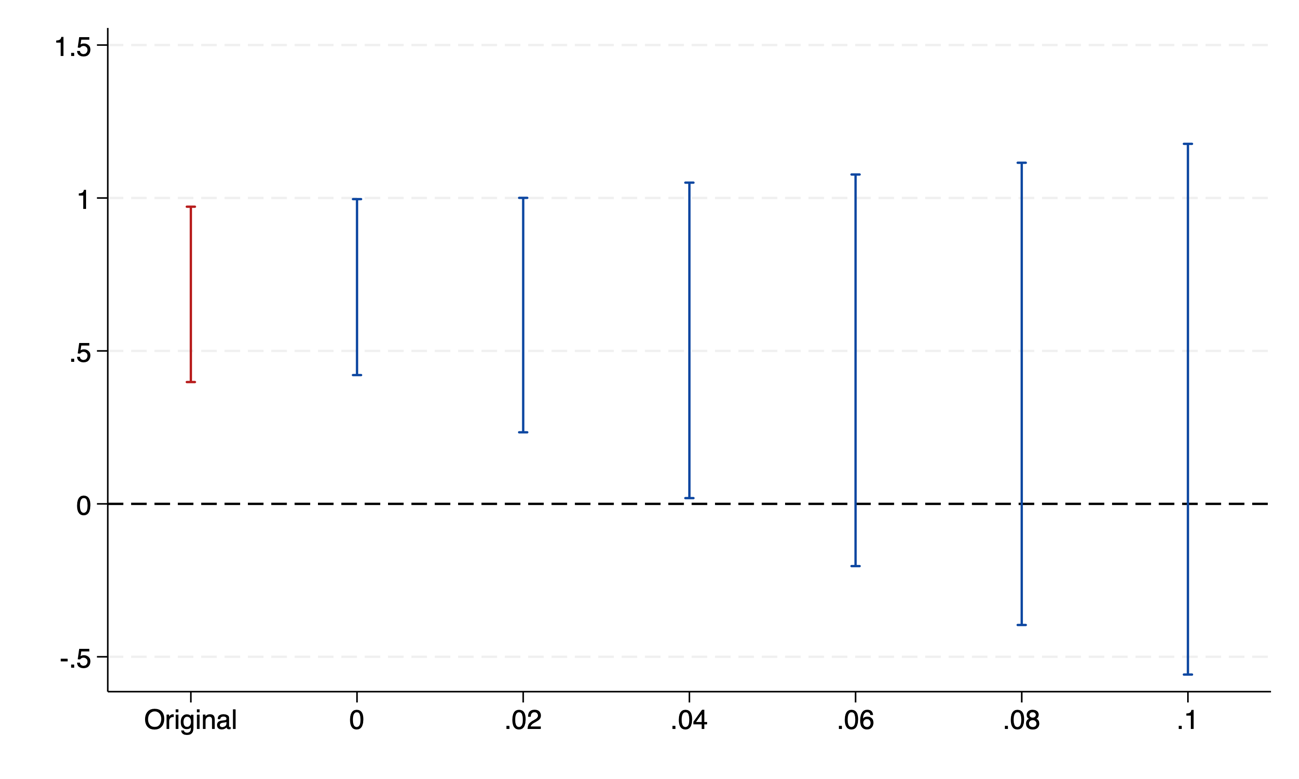


**Fig. 4.E.** **Sensitivity analysis of the average over the four post-treatment periods for the touristic areas within the Bushes and Cacti vegetation type zones: SD restrictions.**

**Appendix F: Robustness check of contamination buffers**

**Table. 1.F. Robustness check of contamination buffer around treated areas.**

|  | 200m (Original) | 400m | 600m | 1000m |
| --- | --- | --- | --- | --- |
|  |  |  |  |  |
| Agriculture | 1.066^***^ | 1.068^***^ | 1.069^***^ | 1.073^***^ |
|  | (0.0366) | (0.0367) | (0.0369) | (0.0372) |
| *N* | 19,230 | 18,807 | 18,357 | 17,511 |
| Bushes & Cacti | 0.685^***^ | 0.684^***^ | 0.697^***^ | 0.687^***^ |
|  | (0.146) | (0.146) | (0.147) | (0.145) |
| *N* | 1,791 | 1,773 | 1,728 | 1,653 |

Robustness estimation of contamination buffer around treated areas. The ATT is reported across all columns, estimated by Doubly Roboust Difference-in-Difference following Callaway & Sant’anna (2021). First column is estimated using original set up of 200m buffer, second column uses a 400m buffer, third one is setted using a 600m buffer, and fourth column works with a 1000m buffer. Standard errors in parentheses.

^*^ *p* < 0.05, ^**^ *p* < 0.01, ^***^ *p* < 0.001

**Appendix G -- Machine Learning Estimations of Treatment Effects.**

We test the robustness of our results using machine learning methods to estimate the $ATT_{t}$. Specifically, we apply the supervised machine learning algorithm proposed by Chernozhukov et al. (2018) [32] to flexibly control for covariates measured in the base period using different learners and stacking them.

We consider six learners for $E\left[ {\Delta Y}_{i} \right|X_{i},T_{i}]$: (i) OLS regression controlling for rainfall, temperature and elevation; (ii) OLS regression controlling for cubic functions of rainfall, temperature and elevation; (iii) Lasso regression controlling for cubic functions of rainfall, temperature and elevation; (iv) Ridge regression controlling for cubic functions of rainfall, temperature and elevation; (v) a random forest using cubic functions of rainfall, temperature and elevation with a minimum 3 observations per leaf; (vi) a random forest using cubic functions of rainfall, temperature and elevation with a minimum 20 observations per leaf.

Similarly, for $E\left[ T_{i} \right|X_{i}]$ we use the following learners: logit regression controlling for rainfall, temperature and elevation; logit regression controlling for cubic functions of rainfall, temperature and elevation; Lasso regression controlling for cubic functions of rainfall, temperature and elevation; Ridge regression controlling for cubic functions of rainfall, temperature and elevation; a random forest using cubic functions of rainfall, temperature and elevation with minimum 3 observations per leaf; and a random forest using cubic functions of rainfall, temperature and elevation with minimum 20 observations per leaf.

We estimate each year separately using the DDML Stata Command (Ahrens et al. 2024) [31]. We consider 5 cross-fit folds and report the median across 10 resamples.


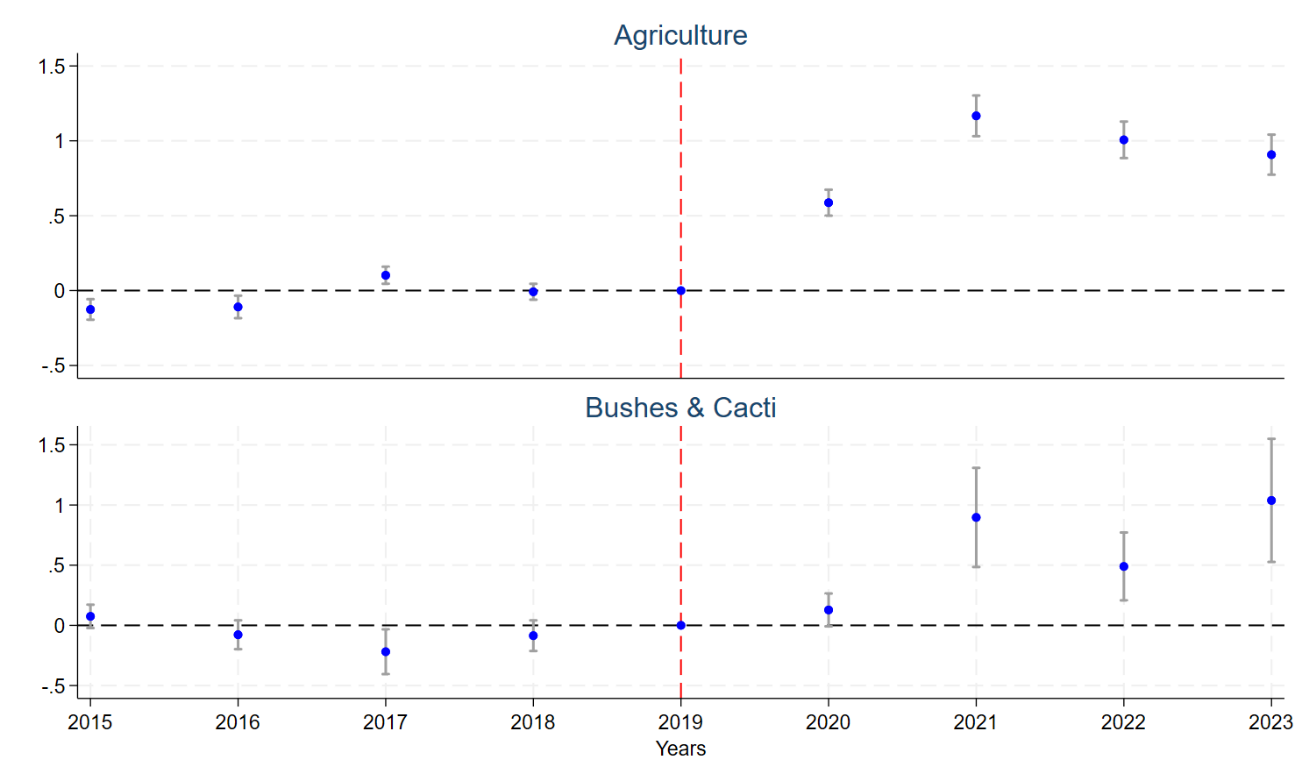


**Fig. 1.G.** **Effects on the LAI across vegetation type zones using Machine Learning methods.**

**Appendix H – Robustness Check of Temporal Aggregation**

**Table. 1.H. Robustness check using monthly aggregated data**

|  | Agriculture | Bushes and Cacti |
| --- | --- | --- |
| ATT | 1.414^***^ | 0.969^***^ |
|  | (0.0649) | (0.264) |
| Weather Controls | X | X |
| Altitude | X | X |
| Observations | 218010 | 20636 |
| Number of Grid cells | 2137 | 215 |
| Treated Cells | 389 | 21 |
| Control Cells | 1748 | 194 |
| Periods | 108 | 108 |

Differences in Differences estimation for monthly aggregated data, with 1000m Hexagonal grids, using Callaway-Sant'Anna (2021). The treatment begins in March of 2020 (month 61 in the data). Estimation was made with Altitude and Weather controls. Errors are clustered at grid-cell level

^*^ *p* < 0.05, ^**^ *p* < 0.01, ^***^ *p* < 0.001

**Appendix I – Robustness to Land-Use Classification Thresholds**

**Table. I.1. Robustness Check: Alternative Land-Use Classification Thresholds**

| Panel A: Agricultural Areas | | | | | |
| --- | --- | --- | --- | --- | --- |
|  | 90% | 80% (original) | 70% | 60% | 40% |
| ATT | 1.085*** | 1.066*** | 1.059*** | 1.045*** | 1.042*** |
|  | (0.0377) | (0.0366) | (0.0359) | (0.0355) | (0.0352) |
| Weather Controls | X | X | X | X | X |
| Altitude | X | X | X | X | X |
| Observations | 16767 | 19230 | 21150 | 23235 | 26310 |
| Number of Grid cells | 1863 | 2137 | 2351 | 2583 | 2926 |
| Treated Cells | 389 | 389 | 389 | 389 | 389 |
| Control Cells | 1474 | 1748 | 1962 | 2194 | 2537 |
| Periods | 9 | 9 | 9 | 9 | 9 |
| Panel B: Tourism – Bushes & Cacti Areas | | | | | |
|  | 90% | 80% (original) | 70% | 60% | 40% |
| ATT | 0.685*** | 0.685*** | 0.685*** | 0.685*** | 0.685*** |
|  | (0.146) | (0.146) | (0.146) | (0.146) | (0.146) |
| Weather Controls | X | X | X | X | X |
| Altitude | X | X | X | X | X |
| Observations | 1791 | 1791 | 1791 | 1791 | 1791 |
| Number of Grid cells | 215 | 215 | 215 | 215 | 215 |
| Treated Cells | 21 | 21 | 21 | 21 | 21 |
| Control Cells | 194 | 194 | 194 | 194 | 194 |
| Periods | 9 | 9 | 9 | 9 | 9 |

DiD estimation using Callaway-Sant'Anna (2021) with 1000m Hexagonal grids. Altitude and Weather controls. Errors clustered at grid-cell level. The rule for assigning control units to the agriculture group is redefined in each column, from 90% in column 1, to 40% in column 5. Original estimations are performed in column 2. Panel A is for Agricultural estimations, Panel B is for Bushes & Cacti toursim areas.

* p<.1, ** p<.05, *** p<.01

**Appendix J – Robustness Check for Grid Dimensions**

**Table. J.1. Robustness Check for Grid Dimensions over Agricultural Areas**

|  | Hexagonal Grids | | Squared Grids |
| --- | --- | --- | --- |
|  | 1000m (original) | 500m | 300m |
| Agricultural ATT | 1.066^***^ | 0.978^***^ | 0.917^***^ |
|  | (0.0366) | (0.0206) | (0.0137) |
| Weather Controls | X | X | X |
| Altitude | X | X | X |
| Number of Grid cells | 2137 | 8649 | 20716 |

**Table J.2. Robustness Check for Grid Dimensions over Touristic Areas (Bushes & Cacti)**

|  | Hexagonal Grids | | Squared Grids |
| --- | --- | --- | --- |
|  | 1000m (original) | 500m | 300m |
| Bushes & Cacti ATT | 0.685^***^ | 0.607^***^ | 0.598^***^ |
|  | (0.146) | (0.0750) | (0.0520) |
| Weather Controls | X | X | X |
| Altitude | X | X | X |
| Number of Grid cells | 215 | 867 | 2080 |

Difference-in-Differences estimations based on yearly aggregated data at different spatial resolutions (1000 m and 500 m hexagonal grids; 300 m square grids). Main specifications follow Callaway and Sant’Anna (2021) for Agriculture and Bushes & Cacti outcomes. Models include altitude and weather controls, with standard errors clustered at the grid-cell level. Treatment and control areas are defined as in the original design to avoid contamination and switching across groups.

**Appendix K – Robustness check for varied tourism area identification**

Table. 1.K Estimation Results using varied area identification

|  | 300m | 500m (original) | 750m | 1000m |
| --- | --- | --- | --- | --- |
| Bushes & Cacti ATT | 0.684^***^ | 0.685^***^ | 0.689^***^ | 0.556^***^ |
|  | (0.152) | (0.146) | (0.147) | (0.126) |
| Weather Controls | X | X | X | X |
| Altitude | X | X | X | X |
| Number of Grid cells | 213 | 215 | 210 | 208 |
| Control Grid cells | 19 | 21 | 21 | 27 |
| Treated Grid cells | 194 | 194 | 189 | 181 |

Differences in Differences estimation for yearly aggregated data, with 1000m hexagonal grids, using Callaway-Sant'Anna (2021), for Agricultural and Bushes and Cacti Areas (main estimations). The specifications remain the same while the buffer’s distance from trails, roads, and tourism spots changes between 300m, 500m, 750m, and 1000m around the shapes. Estimation was made with Altitude and Weather controls. Errors are clustered at grid-cell level.

^*^ *p* < 0.05, ^**^ *p* < 0.01, ^***^ *p* < 0.001
